# Genome-Wide Association Study for Spot Blotch Resistance in Hard Winter Wheat

**DOI:** 10.3389/fpls.2018.00926

**Published:** 2018-07-06

**Authors:** Girma T. Ayana, Shaukat Ali, Jagdeep S. Sidhu, Jose L. Gonzalez Hernandez, Brent Turnipseed, Sunish K. Sehgal

**Affiliations:** Department of Agronomy, Horticulture, and Plant Science, South Dakota State University, Brookings, SD, United States

**Keywords:** *Triticum aestivum*, hard winter wheat, spot blotch, GWAS, QTLs, SNPs, marker-assisted selection

## Abstract

Spot blotch (SB) caused by *Cochliobolus sativus* (anamorph: *Bipolaris sorokiniana*) is an economically important disease of wheat worldwide. Under a severe epidemic condition, the disease can cause yield losses up to 70%. Previous approaches like bi-parental mapping for identifying SB resistant genes/QTLs exploited only a limited portion of the available genetic diversity with a lower capacity to detect polygenic traits, and had a lower marker density. In this study, we performed genome-wide association study (GWAS) for SB resistance in hard winter wheat association mapping panel (HWWAMP) of 294 genotypes. The HWWAMP was evaluated for response to *B. sorokiniana* (isolate SD40), and a range of reactions was observed with 10 resistant, 38 moderately resistant, 120 moderately resistant- moderately susceptible, 111 moderately susceptible, and 15 susceptible genotypes. GWAS using 15,590 high-quality SNPs and 294 genotypes we identified six QTLs (*p* = <0.001) on chromosomes 2D, 3A, 4A, 4B, 5A, and 7B that collectively explained 30% of the total variation for SB resistance. Highly associated SNPs were identified for all six QTLs, *QSb.sdsu-2D.1* (SNP: Kukri_c31121_1460, *R*^2^ = 4%), *QSb.sdsu-3A.1* (SNP: Excalibur_c46082_440, *R*^2^ = 4%), *QSb.sdsu-4A.*1 (SNP: IWA8475, *R*^2^ = 5.5%), *QSb.sdsu-4B.1* (SNP: Excalibur_rep_c79414_306, *R*^2^ = 4%), *QSb.sdsu-5A.1* (SNP: Kukri_rep_c104877_2166, *R*^2^ = 6%), and *QSb.sdsu-7B.1* (SNP: TA005844-0160, *R*^2^ = 6%). Our study not only validates three (2D, 5A, and 7B) genomic regions identified in previous studies but also provides highly associated SNP markers for marker assisted selection. In addition, we identified three novel QTLs (*QSb.sdsu-3A.1*, *QSb.sdsu-4A.*1, and *QSb.sdsu-4B.1*) for SB resistance in wheat. Gene annotation analysis of the candidate regions identified nine NBS-LRR and 38 other plant defense-related protein families across multiple QTLs, and these could be used for fine mapping and further characterization of SB resistance in wheat. Comparative analysis with barley indicated the SB resistance locus on wheat chromosomes 2D, 3A, 5A, and 7B identified in our study are syntenic to the previously identified SB resistance locus on chromosomes 2H, 3H, 5H, and 7H in barley. The 10 highly resistant genotypes and SNP markers identified in our study could be very useful resources for breeding of SB resistance in wheat.

## Introduction

Wheat (*Triticum aestivum* L) is an important cereal crop grown worldwide and remains a vital source for human food (FAO, 2017). However, its production is continuously challenged by a number of environmental and biological factors (Sharma et al., 2007b; Gupta et al., 2018). Spot blotch (SB) caused by *Bipolaris sorokiniana* (Sacc.) Shoem syn. *Drechslera sorokiniana* (Sacc.) Subrm and Jain (syn. *Helminthosporium sativum*, teleomorph *Cochliobolus sativus*) is one of the destructive fungal diseases that affects wheat and several other small grains worldwide (Dubin and Rajaram, 1996; Duveiller and Dubin, 2002; Joshi and Chand, 2002; Sharma et al., 2007b; Singh and Singh, 2007; Gurung et al., 2009; Chowdhury et al., 2013). It has a wide range of hosts within wild and cultivated *Poaceae* species (Kumar et al., 2002; Pandey et al., 2005; O’Boyle et al., 2014). In susceptible lines, SB symptoms are characterized by small, dark brown lesions that extend 1–2 mm long without chlorotic margins during initial infection (Chand et al., 2003). Later, the leaves are killed when the light brown to dark brown colored oval to elongated blotches extend and merge very quickly. In addition to leaves, the fungus causes common root rot (Wildermuth et al., 1997), seedling blight and seed rot or black point on the embryo (Kumar et al., 2002; Hudec and Muchova, 2008). Average yield loss of 15–20% due to SB has been reported from several countries, but under suitable climatic conditions the losses in yield can reach up to 70% in susceptible genotypes, in addition to the reduction in seed quality (Mehta et al., 1992; Lemerle et al., 1996; Fernandez et al., 1998, 2014; Wang et al., 2002; Fernandez and Jefferson, 2004; Sharma et al., 2004, 2007b; Siddique et al., 2006; Sharma and Duveiller, 2007; Acharya et al., 2011).

Breeding for resistance is one of the most economical and sustainable component of integrated disease management (Crute and Pink, 1996; Bartoš et al., 2002; Duveiller et al., 2007; Gupta et al., 2010; Khan et al., 2010; Chowdhury et al., 2013; Ban et al., 2016; Vasistha et al., 2016; Kumar et al., 2017). However, our ability to develop SB resistant genotypes depends on an understanding of the mechanism of resistance present in the host and identification of the resistant genes responsible for SB resistance. Given the challenges in large-scale germplasm screening (Leng et al., 2016; Osman et al., 2016; Kumar et al., 2017), development of molecular markers linked to disease resistance genes can facilitate marker-assisted selection (MAS) and increase the efficiency of breeding for disease resistance in wheat (Collard et al., 2005; Gupta et al., 2010; Miedaner and Korzun, 2012; Müller et al., 2018).

With the availability of large numbers of molecular markers (Miedaner and Korzun, 2012; Korte and Farlow, 2013) more efficient mapping techniques like genome-wide association studies (GWAS) have become popular for analyzing an unlimited number of traits in genetically identical materials across a wide range of environments (Gupta et al., 2005; Ersoz et al., 2007; Miedaner and Korzun, 2012; Korte and Farlow, 2013; Ogura and Busch, 2015; Kushwaha et al., 2017). GWAS has been used to characterize disease resistance in many crop species: blast resistance genes in rice (Raboin et al., 2016), maize (Xiao et al., 2017), SB resistance in wild barley (Roy et al., 2010), resistance to multiple leaf spot diseases of spring wheat (Gurung et al., 2014), resistance to bacterial leaf streak and SB in spring wheat (Adhikari et al., 2012), *Fusarium* head blight resistance in wheat (Arruda et al., 2016), tan spot resistance in European winter wheat (Kollers et al., 2014), mapping for resistance to leaf and stripe rust in winter-habit hexaploid wheat landraces (Sun et al., 2015).

Complex quantitative inheritance (Dubin and Rajaram, 1996; Joshi et al., 2004b; Kumar et al., 2015b) of SB resistance in wheat has slowed the progress in breeding for SB resistance. Many studies, using methods of both bi-parental mapping and association mapping (AM) have reported several SB resistance QTLs on chromosome 1A, 1B, 2A, 2B, 2D, 3B, 5A, 5B, 6B, 6D, 7A, 7B, and 7D (Neupane et al., 2007; Sharma et al., 2007a; Gonzalez-Hernandez et al., 2009; Kumar et al., 2009, 2010, 2015a, 2017; Adhikari et al., 2012; Lillemo et al., 2013; Zhuang et al., 2013; Gurung et al., 2014; Zhu et al., 2014; Lu et al., 2016; Zhang et al., 2015; Gupta et al., 2018). However, only three major QTLs designated as *Sb1* on 7D (Lillemo et al., 2013), *Sb2* on 5B (Kumar et al., 2015a), and *Sb3* on 3B (Lu et al., 2016) are well described. Most of these studies have been focused on hard spring wheat, and relatively few studies characterized SB resistance in hard winter wheat germplasm.

Our ability to deploy and develop SB resistant winter wheat cultivars depends on identification of resistant QTLs responsible for the traits. Therefore, the objectives of this study were (i) to identify winter wheat genotypes carrying resistance genes against *B. sorokiniana* and (ii) to locate putative QTLs and identify SNP markers useful for MAS. This work will contribute toward the development of genome-wide breeding strategies and MAS for SB resistance in wheat.

## Materials and Methods

### Plant Materials

A set of 294 genotypes (Supplementary Table 1) from the hard winter wheat association mapping panel (HWWAMP) of 299 genotypes developed under the USDA TCAP project (Guttieri et al., 2015, 2017) were selected for this study due to availability of sufficient seed. The genotypes were comprised of landraces, advanced breeding lines, and varieties released since the 1940s representing United States Great Plains areas. The HWWAMP panel was designed to include genotypes that represent the existing germplasm of the wheat growing regions of the United States such as Colorado, Kansas, Michigan, Montana, Nebraska, North Dakota, Oklahoma, South Dakota, and Texas (Grogan et al., 2016; Guttieri et al., 2017).

### Evaluating HWWAMP for Spot Blotch

The screening of 294 genotypes and the susceptible check, Glenlea and resistant check, Salamouni against *B. sorokiniana* was conducted at the South Dakota State University Young Brothers Seed Technology greenhouse complex in Brookings, SD, United States. The experiment was conducted in a randomized complete block design (RCBD) with three replications and repeated three times. Three seeds of each genotype were planted in a single root trainer container (Ray Leach “Cone-tainer”^TM^ Single Cell System) and arranged within each tray (Stuewe & Sons, Tangent, OR, United States). Plants were grown in greenhouse at 24°C/16°C (day/night) with 14-h photoperiod and 32% relative humidity. Soluble fertilizer was added every 2 weeks after planting and watering was scheduled every 2 days.

One of the *B. sorokiniana* isolates, SD40, was used as inoculum in all experiments. SD40 is one of the most virulent isolate predominantly found in United States Great Plains areas and routinely used to screen breeding materials (provided by Dr. Shaukat Ali, SDSU). The isolate was originally derived from a single spore and method of its isolation and cultivation is well described by Kumar et al. (2007). This fungus is easily identifiable based on the color morphology and number of nuclei in lab conditions (Shoemaker, 1959). The stored conidia of the fungus were streaked on V8-PDA (Lamari and Bernier, 1989) plates using a sterile rod. The cultures were grown at room temperature under continuous darkness and harvested 5 days later when the pink colony in the plate began to darken. The spores in the plates were scraped with a flamed microscope slide, diluted with distilled water and transferred to beaker covered by cheesecloth to filter out mycelium. The spore suspension was adjusted to 3500/ml prior to use.

A fully expanded third-leaf of all the seedlings were sprayed with spore suspension using a hand held sprayer (Preval, Chicago Aerosol, Coal City, IL, United States). The spore suspension for inoculations was prepared by adding a 100 μl/L Tween-20 (polyoxyethylene-20-sorbitan monolaurate (Sigma-Aldrich, St. Louis, MO, United States) as a dispersing agent. Approximately 0.5 ml of inoculum suspension was applied to each plant using the sprayer. After inoculation, seedlings were incubated in darkness for 12 h at 20°C in a mist chamber near 100% RH by setting the humidifiers to release the mist for 2 min every 30 min to maintain a humid environment for disease development. Subsequently, plants were transferred to the greenhouse bench where the temperature was regulated at 24°C/16°C (day/night) until the plants were rated for symptom development 10 days post-inoculation.

The infection responses (IRs) of each genotype for SB were assessed based on the five-class (1–5) rating scale used by Lamari and Bernier (1989). IRs were based on the type (presence of necrosis and/or chlorosis) and relative size of lesions observed on the third leaves of the seedlings (Supplementary Figure 1). The third fully expanded inoculated leaf during inoculation was rated as follows: (1) small dark brown to black spot without any surrounding chlorosis or necrosis (resistant), (2) small dark brown to black spot with very little necrosis or chlorosis (moderately resistant), (3) small dark brown to black spot completely surrounded by distinct chlorosis or tan necrosis ring, lesions generally not coalescing (moderately resistant to moderately susceptible), (4) small dark brown to black spot completely surrounded by distinct chlorotic or tan necrotic zone and some of the lesions coalescing (moderately susceptible), and (5) the dark brown or black centers may not be distinguishable, most lesions consist of coalescing chlorotic or tan necrotic zones (susceptible). The infection type corresponding to each score in our experiment is shown in Supplementary Figure 1. In our study, we rated the plants based on necrosis symptoms as SB does not produce chlorotic symptoms in wheat.

### Statistical Analysis

Distribution of the phenotypic data for SB was visualized using the histogram. The Shapiro–Wilk test was conducted in R to check the normality of the untransformed and transformed data based on square root method. The homogeneity of the variance across experiments was checked using Bartlett’s test. Data from three repeated experiments, each with three replications, were combined and the overall mean of all experiments was used for analysis of GWAS if the experiment was homogenous. The phenotype data was analyzed using linear mixed model (LMM) approaches with a randomized group-based jackknife technique using R version 3.3.3 (R Core Team, 2016). Broad sense heritability (*H*^2^) was estimated by dividing genetic variance over the combined sum of error variance and genetic variance.

The genotype data were obtained from the wheat T3 Toolbox, a public repository.^1^ The genotyping was conducted using Illumina iSelect 90K under the USDA-TCAP (Guttieri et al., 2017). To avoid spurious marker-trait associations, SNP markers with minor allele frequency (MAF) <0.05 and missing data >10% were excluded from further analyses. The genetic and physical positions of SNP markers from the wheat 90K array were obtained from the consensus map with 46,977 SNPs developed using a combination of eight mapping populations (Wang S. et al., 2014) and the International Wheat Genome Sequencing Consortium website.^2^ After filtering the high-quality polymorphic SNPs, markers were imputed using TASSEL Version 5.0 software (Bradbury et al., 2007).

### Population Structure and Kinship

In order to avoid the distortion of population structure and linkage disequilibrium (LD), SNP markers were first thinned into 0.0005 cM apart to retain only markers with high pairwise correlation using TASSEL v5 software (Bradbury et al., 2007). After keeping informative SNPs in the analysis and eliminating redundant information, we analyzed the genetic stratification, i.e., population structure (Q) within the HWWAMP with STRUCTURE v2.3.4 (Pritchard et al., 2000) using a model-based clustering method. STRUCTURE runs were performed for each specified *K* values (number of subpopulations, from 2 to 6) using the default setting of the admixture model for the ancestry of individuals and correlated allele frequencies. Burn-in period and a number of Markov Chain Monte Carlo (MCMC) iterations under Linux environment were set to 20,000 and 50,000, respectively (Evanno et al., 2005). The best fit number of clusters was calculated according to Evanno et al. (2005) using STRUCTURE HARVESTER (Duncan et al., 2017). The likely number of population structure was chosen from principal coordinates (PCO) plot, i.e., *K* vs. Δ*K* where the rate of change in the log probability between successive *K* values was the highest.

### Linkage Disequilibrium Estimation

Linkage disequilibrium is defined as the non-random association of alleles at different loci in a given population and is represented by the square of the correlation coefficient (*r^2^*) between markers. Markers which were in perfect LD (*r^2^* = 1) with another markers were removed before the LD analysis. The *r^2^* between intra- and inter-chromosomal SNP markers were estimated using TASSEL v5 (Bradbury et al., 2007). LD estimates expressed as *r^2^* and based on a sliding window of 100 markers throughout the genome, were calculated and plotted against genetic distance. From the unlinked loci, two markers were considered significant when LD *P* < 0.001. We plotted the intra-chromosomal *r^2^* values against the genetic distance using excel to see how rapidly the LD decay occurs. The distance at which the smooth curve intercepts the critical *r^2^* was drawn using logarithmic trended smooth lines as described by Hao et al., 2012. A critical value of *r^2^* (basal LD) was estimated using 95% percentile of non-syntenic (inter-chromosomal) *r^2^* distribution below which relationship between two pairs of loci are assumed not to be caused by physical linkage (Laidò et al., 2014). The distance at which the LD decays to 0.7 cM was considered as the critical distance up to which a QTL region can extend (Zhao et al., 2005).

### Genome-Wide Association Analysis

Genomic regions associated with SB resistance were identified using TASSEL v.5.0 (Bradbury et al., 2007) and the new enhanced version of the genome association and prediction integrated tool (GAPIT) (Tang et al., 2016) in R version 3.3.3 (R Core Team, 2016). The enhanced version of GAPIT implements computationally powerful statistical approaches such as general linear model (GLM), mixed linear model (MLM) (Zhang Z. et al., 2010), compressed mixed linear model (CMLM) (Li et al., 2014), enhanced compressed mixed linear model (ECMLM) (Li et al., 2014), factored spectrally transformed LMMs (FaST-LMM Select) (Listgarten et al., 2013) and SUPER (Settlement of MLM Under Progressively Exclusive Relationship) (Wang Q. et al., 2014). In GLMs, marker data, disease data and the PCA matrix were integrated as covariates to correct for the effects of population substructure. Unlike GLM, MLM accounts for both population structure and individual kinship as a covariate to reduce type-I error.

We selected the MLM method for our data by comparing the statistical power they have and type I error they produce (results not presented). The MLM for GWAS were represented by *y* = *X*β + *Qv* + *Ku* +*e*, where *y* is the vector of the phenotypic values, β is fixed effects due to marker, *v* is fixed effects due to population structures, *e* is the vector of residuals, and *u* is a vector of random effects due to the portion of breeding values not accounted by the marker. *X*, *Q*, and *K* represent matrices from the marker, population structure estimated from the structure or principal component analysis and kinship, respectively. The variance of *u* is derived as, Var (*u*) = 2 *KVg*, where *K* represents the relative kinship matrix inferred from genotypes of the HWWAMP based on the proportion of shared alleles and *Vg* is the additive portion of the genetic variance.

### *In Silico* Annotation of SNPs and Syntenic Regions

The sequences of the significant markers associated with SB were extracted from the Infinium iSELECT 90K (Cavanagh et al., 2013) and were BLASTN searched against the Chinese Spring wheat RefSeq v1.0 (IWGSC 2018^3^). The search was limited to the top hit with an *E*-value cut off of 1E-50 with an identity higher than 75%. Being allohexaploid species (2*n* = 6*x* = 42) wheat chromosomes are found in homeologous status (A, B, D) which shared similarities among the homeologous chromosomes. Therefore, we removed SNPs that were mapped to multiple chromosomes.

We further identified the target region for each of the QTLs on pseudomolecule that co-localized with the significant markers contained in each LD block. Next, the sequence segments were BLASTN searched against the wheat coding DNA sequence (CDS) and followed by BLASTX search against the wheat protein database (Duncan et al., 2017). Out of several lists of an annotated protein family, those related to previously described disease resistance protein families were further identified by a BLASTP search against Pfam database (Pfam 31.0^4^). We then compared the candidate regions with barley for comparative analysis of SB resistance genes across related species. We used flanking markers from QTLs identified in our study to BLASTN search against the known barley SB resistance QTLs (Roy et al., 2010; Zhou and Steffenson, 2013) and placed them on the most recent barley genome assembly (Mascher et al., 2017) and then produced synteny representation using circos (Krzywinski et al., 2009).

## Results

### Variations in Seedling Infection Response

**FIGURE 1:**
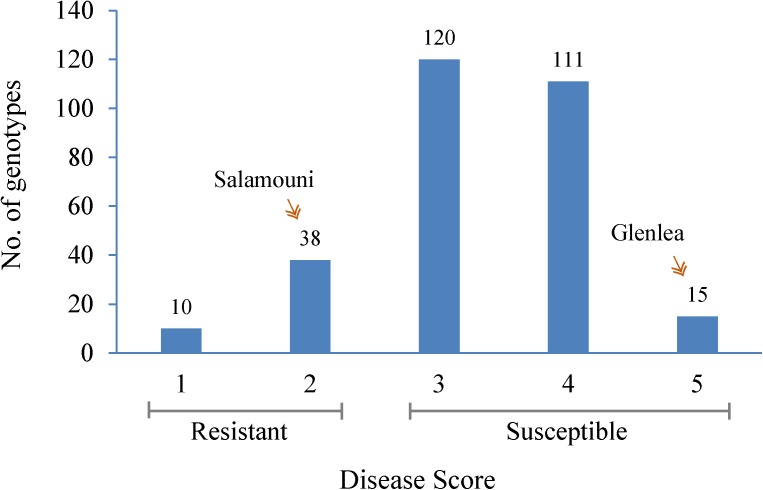
Frequency distribution of the mean spot blotch infection response of 294 HWWAMP genotypes. The *x*-axis exhibits 1–5 scores of mean infection response of each genotype. The *y*-axis represents the number of genotypes (also numbered on the bar) that exhibited the infection response. Salamouni and Glenlea were the resistant and susceptible checks of the experiment, respectively.

Seedlings showed a range of infection types (Supplementary Figure 1) within 294 hard winter wheat genotypes when inoculated with *B. sorokiniana* (**Figure 1** and Supplementary Table 1). As expected, the resistant check, Salamouni and susceptible check, Glenlea exhibited a mean disease score of 2 and 5, respectively (**Figure 1**). Out of 294 genotypes, a total of 48 were resistant whereas other 240 genotypes appeared to be in the moderately resistant – moderately susceptible, moderately susceptible and susceptible categories in all three experiments. Out of the 48 resistant genotypes, 10 showed highly resistant (score 1) and 38 showed modrately resistant reaction (score 2) to the SB. The 10 highly resistant genotypes could be potential sources for SB resistance breeding (**Table 1**). Of the other 248 accessions, 120 showed either moderately resistant – moderately susceptible (score 3) response, whereas, 111 genotypes showed a moderately susceptible (score 4) and 15 showed a highly susceptible (score 5) response to SB of wheat (**Figure 1**).

**Table 1:** List of 10 genotypes from the HWWAMP that showed highly resistant reactions (R) against *B. sorokiniana*.

Genotype	Pedigree	Source	Year released	SB score
Duster	W0405D/NE78488//W7469C/ TX81V6187	OK	2006	1
Colt	Agate sib (NE69441)//(Tx65A1503-1) 391-56-D8/Kaw	NE	1983	1
Custer	F-29-76/TAM-105//Chisholm	OK	1994	1
Intrada	Rio Blanco/TAM 200	OK	2000	1
MT0495	MT9640/NB1133	MT		1
NE99495	ALLIANCE/KARL 92	NE		1
OK04525	FFR525W/Hickok//Coronado	OK		1
OK05122	KS94U337/NE93427	OK		1
OK05723W	SWM866442/Betty	OK		1
Venango	HBE1066-105/HBF0551-137	KS	2000	1

Cultivars and breeding lines that exhibited a highly resistant response to the SB pathogens were: Duster, Colt, Custer, Intrada, MT0495, NE99495, OK04525, OK05122, OK05723W, and Venango (**Table 1**). The variation among data sets of three different experiments and among the genotypes was analyzed using LMM approaches with a randomized group-based jackknife resampling technique (Supplementary Table 2). At α = 0.05, there were highly significant differences among the predicted genotypic effects (*P* = 1.33e-14) when compared to the population mean (μ) (Supplementary Table 2). On the other hand, the variations among the disease score of repeated experiments were not significant. There was a high repeatability (96%) among the experiments, and the phenotypic variation explained by the genotypes (broad sense heritability *H*^2^) was estimated to be 80% (Supplementary Table 2).

### Genotyping, Marker Distribution and Allele Frequency

We obtained genotypic data for 294 HWWAMP constituting 21,555 SNPs from the T3 wheat database.^5^ We removed 5,487 markers by filtering markers with a (MAF less than 5%. Further, 458 markers with unknown chromosome positions were also removed. Finally, 15,590 high-quality SNP markers across 294 accessions of HWWAMP were used for structure and GWAS analysis (Supplementary Table 3). The map position of 15,590 SNP loci were obtained from Wang S. et al. (2014). The 15,590 SNPs covered map length of 3,661 cM on all 21 wheat chromosomes giving an average interval distance of 0.54 cM (Supplementary Table 3). The number of SNP markers assigned to the A, B, and D chromosomes were 6,211, 7,630, and 1,749, respectively. The individual length chromosomes on genetic map ranged from 119 to 244 cM. The average number of markers per cM on genome A (4.97) and B (6.6) were relatively higher when compared to D genome (1.5) (Supplementary Table 3). The chromosome 4D (52) and 7D (133) harbored the lowest number of informative markers. Each locus was characterized by the presence of major and minor allele with a frequency between 0.50 to 0.95, and 0.05 to 0.50, respectively.

### LD Estimation

Linkage disequilibrium is the non-random co-segregation of alleles at two or more loci on the same chromosome or between loci on different chromosomes. Out of 15,990 markers used for AM, only 1,842 markers were used for LD analysis taking out non-informative markers. Among a total of 91,307 locus pairs detected, 13,076 locus pairs (14.3%) were in LD at *P* < 0.001 of which 7,744 locus pairs (8.5%) were found at *r*^2^ > 0.1 and *P* < 0.001 (Supplementary Table 4). However, the distance at which LD starts decaying depends on meiotic events and/or genetic drifts occured in the population. We estimated the LD decay distances in the whole genome and within each genome of winter wheat association panel using logarithmic trended smooth lines developed from scatter plots of syntenic *r^2^* vs. the genetic distance (cM) between pairs of two-locus (Supplementary Figure 2). We estimated the distance point where LD value (*r^2^*) decreases below 0.1 or half strength of D’ (D’ = 0.5) based on the curve of the nonlinear logarithmic trend line. The LD (*r^2^* > 0.1) decay distance of about 4.5 cM was estimated for the whole genome. Whereas, LD decay distances for individual A, B, and D genomes were approximately 3.4, 3.6, and 14.2 cM, respectively (Supplementary Figure 2).

### Population Stratification and Kinship

In order to avoid distortion due to population structure, SNP markers were first thinned to retain only 1,842 markers that were at least 0.5 cM apart in TASSEL 5. A STRUCTURE analysis indicated the presence of four subpopulations (K1–K4) in the HWWAMP where the four clusters contained 45, 37, 99, and 113 genotypes, respectively (Supplementary Figures 3, 4). Average distances (expected heterozygosity) between individuals within each cluster (K1–K4) were 0.06, 0.17, 0.27, and 0.23, respectively. Similarly, the net nucleotide distance among structures, i.e., the average probability that a pair of alleles were different among K1 vs. K2, K3, K4 were 0.28, 0.19, 0.16; K2 vs. K3, K4 were 0.24, 0.21; and K3 vs. K4 were 0.10. We further divided the individuals based on their inferred ancestry and made a principal component analysis. Individuals within and across different structures shared genetic similarities, and were considered admixed when the cumulative shared ancestry across the structures was above 40% or they retained greater than 60% ancestry within their respective structure. From a PCA analysis, the proportion and cumulative variances of the first four PCA were 0.43, 0.29, 0.28, 0.00 and 0.43, 0.72, 1.00, 1.00, respectively (results not shown). Similarly, a matrix of kinships among individual genotypes was calculated using all 15,990 SNPs. The heat map calculated using the classical equation from VanRaden (2008) showed a high kinship relationship among individuals (Supplementary Figure 5).

### Model Comparison for Marker-Trait Association

Six statistical models such as GLM, MLM, CMLM, ECMLM, FaST-LMM, and SUPER were compared to select the one which reduces the type-I error and increases the power of SNP discovery. Our analysis indicated that MLM, CMLM, and ECMLM similarly reduced the type-I error and increased power when compared to others. The analysis showed no differences in the number of significant SNPs discovered using all the models except GLM and FAST. However, due to its power and robustness, we selected MLM (Zhang Z. et al., 2010) with optimum compression as the model of choice for subsequent analysis. The quantile-quantile (Q–Q) plots for the test statistics (Supplementary Figure 6) indicated the absence of inflation of statistics or overall systematic bias caused by the population stratification when MLM was used than GLM. The number of significant markers associated with the SB response with the GLM were much greater than with the MLM (Supplementary Table 5). Indeed, at *P* < 0.001 there were 13 and 26 significant makers trait associations that were identified by MLM and GLM, respectively. The 13 markers identified by MLM were also significant by GLM (Supplementary Table 5).

### Markers Associated With the Spot Blotch QTLs

**FIGURE 2:**
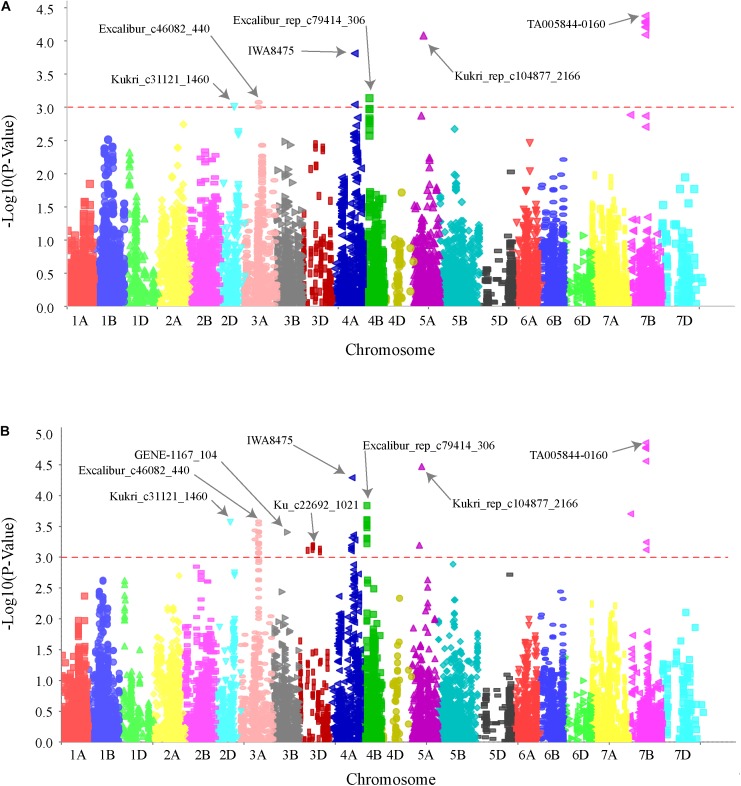
Genome wide association scan for SB resistance in hard winter wheat association mapping panel (HWWAMP). Manhattan plot developed using mixed linear model (MLM) **(A)** and general linear model (GLM) **(B)** in TASSEL v.5. The -log_10_ (P) values from a genome-wide scan are plotted against positions on each of the 21 wheat chromosomes. Horizontal lines indicate genome-wide significance thresholds.

GWAS analysis identified several genomic regions linked to SB resistance including some in genomic regions identified in earlier studies (Joshi et al., 2004a; Lillemo et al., 2013; Kumar et al., 2015a; Lu et al., 2016). We identified six genomic regions showing highly significant (*P* < 0.001) markers associated with SB resistance on chromosomes 2D, 3A, 4A, 4B, 5A, and 7B (**Figure 2**). A total 30% of the variation was explained by the most significant SNP from each genomic region. QTLs for SB resistance have been reported in the regions similar to three of the six QTLs identified in our study on chromosomes 2D (*QSb.sdsu-2D.1*), 5A (*QSb.sdsu-5A.1*), and 7B (*QSb.sdsu-7B.1*). We identified three novel regions that contribute to SB resistance. The QTLs, *QSb.sdsu-3A.1*, *QSb.sdsu-4A.1*, and *Q.Sb.sdsu-4B.1* (*P* < 0.001) explained 4, 6, and 4% of the variation, respectively (**Table 2**). We identified several co-localized SNPs markers that were associated to each QTL region (Supplementary Tables 6, 7). We further identified the QTL spanning region based on the significant SNP markers in LD. The SNP marker Kukri_rep_c104877_2166 which was at 59.1 cM (480,285,174 bp) on chromosome 5A had the highest association (*P* = 4.02E-05 and *R*^2^ = 5.9%). Likewise, group of 7B markers such as Excalibur_c5700_670, Kukri_c21628_1215, Tdurum_contig9966_646, Kukri_c22495_552, Excalibur_c5700_527, Excalibur_c58742_144, TA005844-0160, Excalibur_c5700_705, BS00075332_51, and Tdurum_contig90495_232 were found at about 86.1 cM were high associated with SB resistance and explained similar phenotypic variation (*R*^2^ = 6.3%) (Supplementary Table 6).

**Table 2:** Summary of SNP markers linked to significant SB resistance QTLs detected from genome-wide association analysis of 294 winter wheat genotypes.

No.	QTL (SNP Marker)	Chr.	Alleles	Position (cM)^∗∗^	*p*-value	*R*^2^ (%)	Additive effect^∗^	SB response
1	*QSb.sdsu-2D.1* (Kukri_c31121_1460)	2D	CC/TT	80.2	4.8E^-04^	4.3	-0.45	R
2	*QSb.sdsu-3A.1* (Excalibur_c46082_440)	3A	CC/TT	90.6	9.0E^-04^	4.0	-0.37	R
3	*QSb.sdsu-4A.1* (IWA8475)	4A	GG/TT	118.7	8.7E^-05^	5.5	-0.44	R
4	*QSb.sdsu-4B.1* (Excalibur_rep_c79414_306)	4B	AA/GG	36.8	7.3E^-04^	4.1	+0.38	S
5	*QSb.sdsu-5A.1* (Kukri_rep_c104877_2166)	5A	GG/TT	59.1	3.3E^-05^	6.2	+0.67	S
6	*QSb.sdsu-7B.1* (TA005844-0160)	7B	CC/TT	86.4	3.1E^-05^	6.3	-0.46	R

*R = SD52; S = SD1001; ^∗^Additive effect refers to first allele e.g., for QSb.sdsu-2D.1 it refers to CC. ^∗∗^The cM position is based on Wang S. et al. (2014).*

### QTL Effects on SB Resistance

QTLs with negative additive effect had favorable alleles and showed a reduction in SB whereas QTLs with positive additive effect had alleles showing unfavorable effect on SB reaction (**Table 2** and Supplementary Tables 8, 9). The combination of individual QTL effects produces total resistance effects that the genotype exhibit. The cultivar, Colt, for instance, was one of the most resistant genotypes and harbored desirable alleles on chromosome 2D, 3A, 4A, and 7B. The combination of the four alleles reduced the disease by a score 1.73 when compared to the mean score of the HRWWAMP and hence exhibited the highest resistance (**Table 2** and Supplementary Tables 8, 9). Similarly, OK05723 and Duster had desirable allele on chromosome 4A and 7B, and 3A, 4A, and 7B, respectively, that gave higher protection against SB. QTLs *QSb.sdsu-2D.1, QSb.sdsu-3A.1*, *QSb.sdsu-4A.1*, and *QSb.sdsu-7B.1* could give the highest protection against the disease if a genotype harbored a combination of the following desirable alleles: CC, CC, GG, and CC, respectively (Supplementary Table 9). For QTLs *QSb.sdsu-4B.1* and *QSb.sdsu-5A.1* the best approach would be to have genotypes with alleles AA and TT, respectively (Supplementary Table 9).

### *In Silico* Functional Annotation of the Candidate Region

**FIGURE 3:**
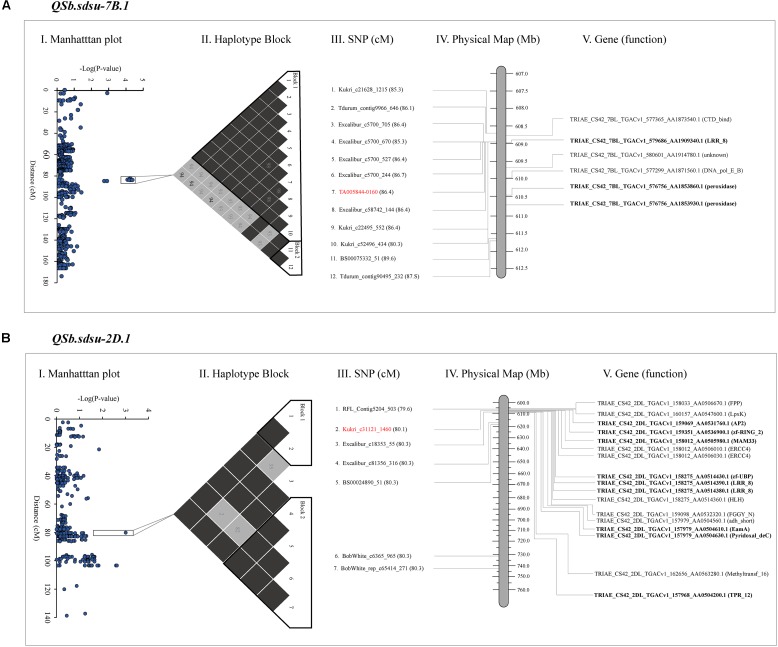
Gene annotation of QTLs identified on chromosome 7B **(A)** and 2D **(B)** for spot blotch resistance in hard winter wheat. The far left image is a Manhattan plot indicating the level of marker association with the trait **(I)**. Next is a visualization of linkage disequilibrium (black is a D’ value of 100%, another color is a D’ value of less than 100%), significant haplotype blocks are outlined in black **(II)**. Names of the markers in the region of interest along with their cM position **(III)**, and physical position **(IV)**. Most significant marker is highlighted in red. The far right image is a physical map of candidate genes on 7B and 2D chromosome segments spanning from 608.7 to 611.7 Mb and 606.9 to 608.8 Mb, respectively (**IV** and **V**). Genes in bold code Pathogenesis-related (PR) proteins. The cM position is based on Wang S. et al. (2014) and the physical position is based on IWGSC 2018.

Plant reactions to diseases are very complex, and involve the activation of sets of genes, encoding for different proteins. To facilitate the identification of additional markers to identify candidate proteins that may be involved in pathogenesis-related (PR) response we annotated the coding sequences in the candidate regions (**Figure 3** and Supplementary Table 10). The chromosome regions spanning through the set of SNP markers that showed *P* < 0.05 up-and downstream of the highest significant marker was identified for further analysis (**Figure 3**). After BLASTN (1e-50) search of markers sequence to reference wheat genome (RefSeq v1.0), a 2.07, 56.7, 6.58, 1.31, 10.03, and 3.00 Mb segment of DNA were identified on chromosomes 2D, 3A, 4A, 4B, 5A, and 7B, respectively and were used for functional annotation. Out of the total of 129 proteins annotated in the candidate regions, we found 9, 22, 5, 2, 9, and 3 disease-related proteins in candidate regions on chromosomes 2D, 3A, 4A, 4B, 5A, and 7B, respectively (Supplementary Table 10). We further studied QTLs *QSb.sdsu-7B.1* and *QSb.sdsu-2D.1* in detail as they explained the maximum variation of SB resistance in our study and were flanked in relatively smaller regions. **Figure 3** shows the SNP associations with SB response and is plotted as Manhattan plot (**Figures 3A-I,B-I**) along the consensus genetic map (Wang S. et al., 2014). Within the selected region of the up-and-downstream of the most significant markers, we compared the local *r^2^* LD pattern and two haplotype blocks were identified on each of chromosome 7B (**Figure 3A-II**) and 2D (**Figure 3B-II**). The haplotype block DNA segment on 7B was 2.6 Mb (1.1 cM) (**Figure 3A-III**) whereas 2D haplotype block was 2.1 Mb (0.7 cM) (**Figure 3B-III**) and the functions of plant disease defense-related genes in the haplotype blocks (**Figures 3A-IV,B-IV**) were identified.

Our gene annotation analysis identified several NB-ARC and NBS-LRR proteins containing *N*-terminal nucleotide-binding site (NBS) and C-terminal leucine-rich repeat (LRRs) in QTLs spanning regions of *QSb.sdsu-2D.1*, *QSb.sdsu-3A.1*, *QSb.sdsu-4A.1*, and *QSb.sdsu-7B.1*. Similarly, Chitinase class I protein was observed in the QTL region *QSb.sdsu-5A.1*. The protein description, functions, ID and other related information are presented in Supplementary Table 10. Proteins related to cell death and response to oxidative stress such as peroxidase (POX) superfamily and zinc-binding dehydrogenase protein family were observed on *QSb.sdsu-7B.1* and *QSb.sdsu-5A.1*, respectively. In addition to NBS-LRR proteins *QSb.sdsu-2D.1* also harbors zinc finger domain and RING/U-box superfamily proteins that may play important role in resistance to fungal pathogens. Further, protein families reported as transporters (EamA-like transporter family), endopeptidase inhibitors (serine protease inhibitor – SERPIN), kinase activity (protein kinase superfamily and tyrosine kinase), and detoxification (ATP-binding cassette (ABC) transporter C and cytochrome P450) were also found in QTL candidate regions. Besides the main families, proteins with unknown function or indirectly involved in disease resistance like senescence and dehydration-associated protein, ABC transporters were identified. Similarly, protein families such as glycosyl hydrolase, mitochondrial glycoprotein, auxin signaling F-box 2 (AFB2), pentatricopeptide repeat (PPR) superfamily, proteins were found in the candidate regions of few QTLs.

### Comparison of SB Resistance QTLs Between Wheat and Barley

**FIGURE 4:**
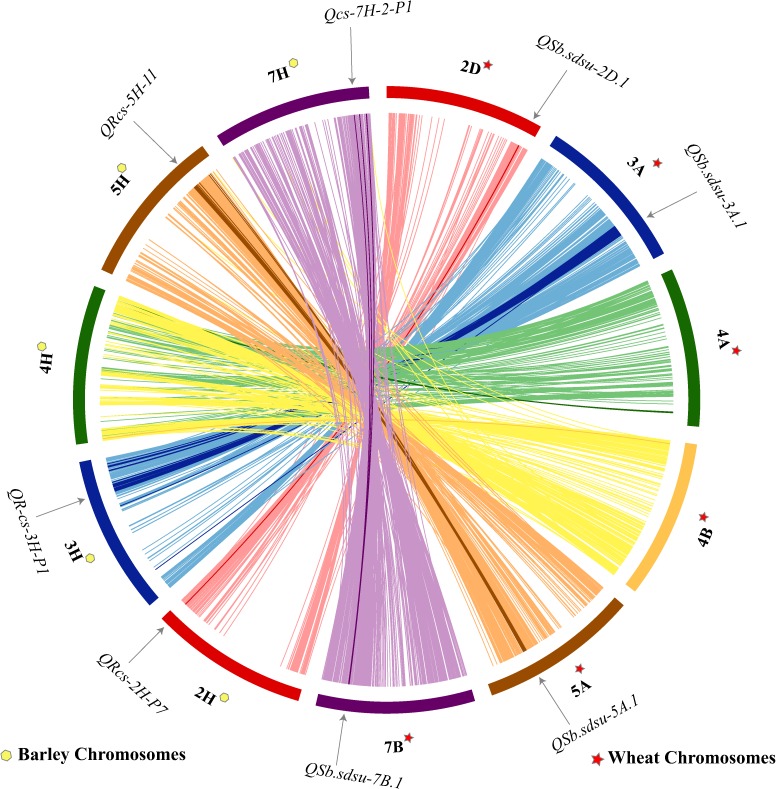
Comparative analysis showing synteny in spot blotch resistance QTLs mapped on wheat (TGACv1 2014) and barley (Mascher et al., 2017) chromosomes. The map was made using CIRCOS (Circular Genome Data Visualization). Each color indicates a different chromosome. Arcs in bold shows the corresponding marker related to the respective QTL.

Shared synteny can be one of the most reliable criteria for establishing the orthology of genomic regions in different species. We performed a comparative analysis of wheat and barley for the candidate regions of six QTLs identified in our study (**Figure 4**). Synteny analysis indicated that four QTLs (*QSb.sdsu-2D.1*, *QSb.sdsu-3A.1*, *QSb.sdsu-5A.1*, and *QSb.sdsu-7B.1*) on chromosomes 2D, 3A, 5A, and 7B carrying SB resistance QTLs in wheat corresponds to 2H, 3H, 5H, and 7H chromosomes in barley (**Figure 4**). SB resistance genes/QTLs have been reported in these syntenic regions in barley (Roy et al., 2010; Zhou and Steffenson, 2013) further validating the QTLs identified in our study.

## Discussion

### Phenotypic Variability for Resistance Against Spot Blotch of Wheat

Developing SB resistant wheat cultivars is likely the most economical and durable strategy for minimizing loss due to SB when compared to the overall usage of fungicides (Duveiller and Gilchrist, 1994; Duveiller and Sharma, 2009; McMullen and Adhikari, 2009). Several sources of SB resistance have been reported in the Indian national collection (Kumar et al., 2017), CIMMYT germplasm and derivatives of CIMMYT primary synthetic bread wheat (Mikhailova et al., 2004; Zhu et al., 2014), multi-resistant cultivars from Nepal (Mahto et al., 2011) and some Brazilian varieties (Mehta, 1998). However, most of these studies have been conducted in spring wheat. In winter wheat, modern European winter wheat cultivars and breeding lines (Liatukas and Ruzgas, 2012) have been evaluated, but the United States germplasm has not been extensively exploited. In the present study, the 294 HWW genotypes showed a variable reaction against SB indicating the existence of genetic variability among them similar to the observations reported in spring wheat (Khan and Chowdhury, 2011; Kumar et al., 2017; Ojha et al., 2017). We observed a repeatability of 96% and a heritability of 80% demonstrating the reliability of the SB resistance evaluation under greenhouse conditions. However, studies have shown both positive and negative correlation for response to SB wheat between *in vivo* and *in vitro* conditions (Rosyara et al., 2009; Liatukas and Ruzgas, 2012). We identified 10 genotypes (**Table 1**) highly resistant to SB that can be utilized for developing SB resistance in winter wheat cultivars. Colt [Agate sib: (NE69441)//(Tx65A1503-1) 391-56-D8/Kaw] was one of the top cultivars that exhibited a reproducible and highly resistant reaction to the SB pathogen (**Table 1**). It was among the first semi-dwarf wheat ever released in Nebraska in 1983. Another cultivar Duster, one of the most popular variety in the Oklahoma since 2006, was found to be highly resistant and could be a promising source of SB resistance. It has a pedigree background of W0405D/NE78488//W7469C/TX81V6187. Similarly, Custer which had been a top performer in Oklahoma in 1987, and some of its parents (F-29-76/TAM-105//Chisholm) were among the HWWAMP accessions were also evaluated in this experiment. Both F-29-76 and TAM-105 showed susceptible reaction whereas Chisholm exhibited resistant reaction suggesting that Chisholm served as sources of resistance in Custer. Of the 168 winter wheat cultivars released in the United States since the green revolution of 1970s and evaluated in our study, only 16% of the genotypes showed a resistant and moderately resistant response (Supplementary Table 1). A similar percentage (16%) of the evaluated breeding lines were also resistant and can be employed for SB resistance breeding.

### SNP Marker Distribution and LD Decay

The distribution and density of informative markers reflects the overall genetic richness and diversity of the wheat genome. The D genome had the least average marker density ranging from 0.3–4.2 markers/cM as compared to A (3.9–6.4) and B (3.8–7.2) genomes. Further, the total markers used in for AM from D genome (11.2%) were very small when compared to A (39.8%) and B (48.9%) genomes suggesting a lower genetic diversity and lower level of effective recombination in the D genome as observed in prevoius studies Barnes et al. (2012) and Nielsen et al. (2014). LD is an integral part of AM and the estimation of LD decay distances can help in determining the power of AM. We estimated a critical value of *r^2^* (basal LD = 0.1) from 95th percentile distribution of the inter-chromosomal LD (Francki et al., 2006; Laidò et al., 2014) below which we assumed the absence of physical relationship within pairs of loci and hence they are physically present on different chromosomes. Our results also demonstrated that the D genome had high LD decay distance (14 cM) when compared to A and B genomes (Supplementary Figure 2). Whole genome LD decay distance we observed in HWWAMP was similar to whole genome LD decay observed of in Chinese winter wheat using SSR marker (Chao et al., 2010; Barnes et al., 2012). However, a lower rate of average LD decay (higher distance) was observed in European hexaploid wheat (23 cM) (Nielsen et al., 2014) and US Elite hard red winter wheat (10 cM) at *r^2^* > 0.1(Zhang D. et al., 2010). When comparing the wheat landraces to modern cultivars, the LD decay distance (5 cM) was lower in landraces as compared to modern winter wheat cultivars (5–10 cM) (Hao et al., 2011) suggesting a possible reduction in diversity in the modern wheat cultivars in China. The LD decay analysis in HWWAMP showed variation among genomes and within the genome itself, indicating variability in recombination hot spots, differences in selection pressure imposed on alleles of wheat genomes, and an evidence of the recombination events in past breeding history.

### QTLs for SB Resistance

We identified six genomic locations on chromosomes 2D, 3A, 4A, 4B, 5A, and 7B associated with SB resistance in HWWAMP and the linked SNPs (TA005844-0160, Kukri_rep_c104877_2166, IWA8475, Kukri_c31121_1460, Excalibur_rep_c79414_306, and Excalibur_c46082_440) explained about 30% of the total variation. We compared the MTA with MLM and GLM (**Figure 2**), and observed that GLM analysis exhibited low power and a higher risk of false-positive detection (result not presented). In the GLM, which only accounts for population structure as a covariate, the additive variance and error variance could not be separated as GLM uses maximum compression (compression = *n*) with all taxa as a single group. Unlike GLM, however, MLM (Zhang Z. et al., 2010) takes account of population structure and individual kinship in association analysis to reduce type I error instigated due to relatedness and population structure. We observed similar results with MLM, cMLM (Tang et al., 2016) and ECMLM (Li et al., 2014). All subpopulations showed a good degree of admixture which could be a result of frequently exchanged germplasm among the winter wheat breeding programs (Supplementary Figure 3).

We identified three novel QTLs for SB resistance (*QSb.sdsu-3A.1*, *QSb.sdsu-4A.1*, and *Q.Sb.sdsu-4B.1*) in addition to three regions on chromosomes 2D, 5A, and 7B reported earlier to be significantly associated with SB resistance. Previously, Gurung et al. (2014) reported significant QTLs associated with SB on chromosomes 1B, 5A, 5B, 6B, and 7B, whereas Adhikari et al. (2012) detected QTLs on chromosomes 1A, 3B, 7B, and 7D, and Lillemo et al. (2013) detected QTLs on chromosomes 5B, 7A, and 7D. Similarly, SB resistance QTLs were reported on wheat chromosomes 1B, 3B, and 5A (Zhu et al., 2014); 2AL, 2BS, 5BL, and 6DL (Kumar et al., 2009); 2BS, 2DS, 3BS, 7BS and 7DS (Kumar et al., 2010); 5B, 6A, and 6D (Sharma et al., 2007b) and 7B and 7D on (Ban et al., 2016). With dense marker coverage, we not only validate the QTLs *QSb.sdsu-2D.1, QSb.sdsu-5A.1*, and *QSb.sdsu-7B.1* on chromosomes 2D, 5A, and 7B but also provided highly significant associated SNP markers that could be used for MAS for SB resistance. However, with a limited number of common markers between previous SSR or DArT based studies, comparisons could be distorted and allelism tests will be required to determine which of the QTLs identified in our study have been previously identified.

In our analysis, none of the three major QTLs, *Sb1* (Joshi et al., 2004a; Lillemo et al., 2013), *Sb2* (Kumar et al., 2015a), and *Sb3* (Lu et al., 2016) contributing to SB resistance that have been characterized using simple sequence repeat (SSR) markers were significant at *P* < 0.001 but all these QTLs showed a peak and were significant at *P* < 0.005 suggesting the presence of these QTLs in the HWWAMP. Each of the three QTLs was explaining ∼3% variation in HWWAMP but was lower than was reported in previous studies (Joshi et al., 2004a,b, Lillemo et al., 2013; Kumar et al., 2015a,b; Lu et al., 2016). The high density of marker coverage in our study and the availability of wheat genome sequences permitted comparison with barley (**Figure 4**). Identification of several R gene clusters and mapping of SB QTLs in syntenic regions in barley suggest four wheat QTLs could be orthologous to barley and this information could support the beginning for the search of candidate genes in wheat and understanding the mechanism of SB resistance.

### Functional Annotations of Candidate Regions

Plant defense systems can be categorized into ever existing constitutive defense systems that are triggered by pathogen-associated molecular patterns (PAMPs) or the temporarily induced system that targets to defend an attacked area of the plant. The genes encoding the specificity determinants of effector-triggered immunity are known as resistance (R) genes (DeYoung and Innes, 2006). The production of PR proteins in response to pathogens are the primary mechanisms in induced plant’s self-defense system. Numerous PR proteins have been characterized in recent years, and largely classified into at least 17 protein families and several pathogenesis-related proteins that do not constitute a superfamily of proteins (Dangl and Jones, 2001; Sels et al., 2008). The genomic regions spanning the SB QTLs identified in our study harbored many genes. However, not all genes are equally important in the regulation of quantitative traits like SB resistance. The candidate genes that were commonly found across multiple QTLs reported are more likely the ones that determine the trait (Swamy et al., 2011). In our study, we found NB-ARC and NBS-LRR in many of the annotated QTL regions (**Figure 3** and Supplementary Table 10). The NBS-LRR are the most common R-genes by which highly conserved NBS domains can bind and hydrolyze ATP or GTP, whereas the LRR motif is typically involved in protein-protein interactions and is responsible for recognition specificity (Wan et al., 2012; Zhang et al., 2016).

Peroxidase superfamily protein that plays a role in self-defense (Hiraga et al., 2001) by catalyzing oxidoreduction between H_2_O_2_ and various reductants was one of the classical enzymes that was observed in the *QSb.sdsu-7B.1* region on chromosome 7B. Chitinase class I was observed in the *QSb.sdsu-5A.1* region. Plant chitinases are the proteins that hydrolyzes the *N*-acetylglucosamine polymer chitin and also takes part in pathogenesis related activities (Punja and Zhang, 1993). Another disease related protein family identified in the candidate regions was protein kinase that not only play major role in phosphorylation in plants (Mulekar and Huq, 2013) but are also involved in signal transduction and activation of plant defense mechanisms (Goff and Ramonell, 2007). Many plant defense related genes such as *OsMPK6* in rice (Ueno et al., 2015) and *Pto* in tomato (Martin et al., 1993) have been reported to translate protein kinase proteins. In our study protein kinase protiens were observed in QTL region *QSb.sdsu-3A.1*, *QSb.sdsu-4A.1*, and *QSb.sdsu-5A.1*. Further, the zinc finger proteins identified in some of the candidate regions in our study have been reported to regulate resistance mechanism through its active involvement in sequence-specific binding to DNA/RNA and contribution in protein-protein recognitions (Gupta et al., 2012). All the above described disease-related genes identified in the candidate regions could help in the development of a new markers and further characterization of the SB resistance QTLs in wheat.

### Implications of GWAS for SB Resistance Breeding in Wheat

The ultimate goal of characterizing SB resistance genes is to find closely linked markers for assisting in the selection and further understanding the underlying network of genes and their interactions to achieve resistance responses. This comprehensive understanding will help in developing durable disease resistant cultivars. We identified groups of SNP markers associated with six QTLs that had different levels of effects (**Table 2** and Supplementary Table 6). A few genotypes used in our study encompass multiple resistant alleles and showed highest level of resistance responses (**Table 1** and Supplementary Table 1). Past inheritance studies on resistance to SB suggest polygenic types of resistance that appears to be based on many minor genes with small individual effects (Dubin and Rajaram, 1996; Joshi et al., 2004b; Gurung et al., 2014; Kumar et al., 2015b). Backcrosses of two parents harboring *Qsb.bhu-2A* on chromosome 2A and *Qsb.bhu-5B* on chromosome 5B in suitable parents achieved higher resistance in susceptible cultivar HUW 234 in India (Vasistha et al., 2016). This suggested that assembling multiple desirable alleles for SB QTLs from multiple parents into a single genotype through the use of marker-assisted gene pyramiding could help in improving disease resistance in wheat varieties. We identified that winter wheat cv. Custer (OK) carries desirable alleles for SB QTLs on chromosomes 2D, 3A, 4A, and 7B and also demonstrated a very high level of SB resistance. The significant SNP markers linked to these SB QTLs (**Table 2** and Supplementary Table 7) can be used to develop a Kompetitive Allele Specific PCR (KASP) assay and used for MAS, as it is well evidenced in several crops.

## Conclusion

We identified 10 winter wheat genotypes highly resistant to SB and six genomic regions associated with SB resistance along with tightly linked SNPs. Genotypes with multiple SB resistance QTLs could be used for future breeding and the linked SNP markers could facilitate quick mobilization of the SB resistance QTLs.

## Author Contributions

GA, SA, and SS designed the experiments. GA and SA performed the SB evaluations. GA, JS, and SS performed the data analysis. GA and SS wrote the manuscript. JS, SA, JGH, and BT contributed to the interpretation of results and also critically reviewed the manuscript.

## Conflict of Interest Statement

The authors declare that the research was conducted in the absence of any commercial or financial relationships that could be construed as a potential conflict of interest.

## Footnotes

1 http://triticeaetoolbox.org

2 http://www.wheatgenome.org

3 https://wheat-urgi.versailles.inra.fr/Seq-Repository/Assemblies

4 https://xfam.wordpress.com/2017

5 https://triticeaetoolbox.org/wheat
