# Supplementary material for: Genome-Wide Association Study for Spot Blotch Resistance in Hard Winter Wheat
Source: Front Plant Sci. 2018 Jul 6;9:926. doi: 10.3389/fpls.2018.00926 (PMC6043670; doi:10.3389/fpls.2018.00926)
Supplement: Supplementary file 1 [file Table_1.docx]

Supplementary Table 1. Mean score of spot botch response in 294 winter wheat genotypes from hard winter wheat association mapping panel (HWWAMP) in three separate experiments.

| **Name** | **Mean of Disease** | | | | **Name** | **Mean of Disease** | | | | | | | |  |
| --- | --- | --- | --- | --- | --- | --- | --- | --- | --- | --- | --- | --- | --- | --- |
|  | **Exp.1** | **Exp.2** | **Exp.3** | **Mean** |  | **Exp.1** | | **Exp.2** | | **Exp.3** | | **Mean** | |  |
| 2145 | 3 | 3 | 3 | 3.0 | CO03W054 | 4 | | 4 | | 4 | | 4.0 | |  |
| 2180 | 3 | 4 | 4 | 3.7 | CO04025 | 4 | | 4 | | 3 | | 3.7 | |  |
| 2174-05 | 3 | 3 | 3 | 3.0 | CO04393 | 3 | | 4 | | 3 | | 3.3 | |  |
| ABOVE | 2 | 3 | 3 | 2.7 | CO04499 | 3 | | 4 | | 4 | | 3.7 | |  |
| AGATE | 2 | 2 | 2 | 2.0 | CO04W320 | 4 | | 5 | | 4 | | 4.3 | |  |
| AKRON | 1 | 2 | 2 | 1.7 | CO050337-2 | 4 | | 3 | | 4 | | 3.7 | |  |
| ALICE | 3 | 3 | 3 | 3.0 | CO07W245 | 3 | | 2 | | 3 | | 2.7 | |  |
| ALLIANCE | 1 | 2 | 2 | 1.7 | CO940610 | 3 | | 3 | | 3 | | 3.0 | |  |
| ANTELOPE | 4 | 4 | 4 | 4.0 | COLT | 1 | | 2 | | 1 | | 1.3 | |  |
| ANTON | 4 | 4 | 4 | 4.0 | COMANCHE | 5 | | 4 | | 4 | | 4.3 | |  |
| ARAPAHOE | 4 | 4 | 4 | 4.0 | COSSACK | 4 | | 5 | | 5 | | 4.7 | |  |
| ARLIN | 2 | 2 | 2 | 2.0 | COUGAR | 2 | | 3 | | 3 | | 2.7 | |  |
| AVALANCHE | 3 | 3 | 3 | 3.0 | CREST | 1 | | 2 | | 2 | | 1.7 | |  |
| BAKERS_WHITE | 4 | 3 | 3 | 3.3 | CRIMSON | 4 | | 4 | | 4 | | 4.0 | |  |
| BENNETT | 2 | 2 | 2 | 2.0 | CULVER | 3 | | 4 | | 4 | | 3.7 | |  |
| BIG_SKY | 3 | 3 | 3 | 3.0 | CUSTER | 2 | | 2 | | 2 | | 2.0 | |  |
| BILL_BROWN | 3 | 3 | 3 | 3.0 | CUTTER | 4 | | 4 | | 4 | | 4.0 | |  |
| BILLINGS | 2 | 2 | 2 | 2.0 | DANBY | 4 | | 4 | | 4 | | 4.0 | |  |
| BISON | 4 | 3 | 4 | 3.7 | DARRELL | 4 | | 4 | | 4 | | 4.0 | |  |
| BOND_CL | 4 | 4 | 3 | 3.7 | DAWN | 2 | | 1 | | 2 | | 1.7 | |  |
| BRONZE | 3 | 3 | 3 | 3.0 | DECADE | 3 | | 3 | | 3 | | 3.0 | |  |
| BUCKSKIN | 3 | 3 | 2 | 2.7 | DENALI | 3 | | 3 | | 3 | | 3.0 | |  |
| BURCHETT | 2 | 3 | 3 | 2.7 | DODGE | 1 | | 2 | | 2 | | 1.7 | |  |
| BYRD | 4 | 3 | 3 | 3.3 | DUKE | 4 | | 4 | | 4 | | 4.0 | |  |
| CAMELOT | 2 | 2 | 2 | 2.0 | DUMAS | 4 | | 4 | | 5 | | 4.3 | |  |
| CAPROCK | 2 | 3 | 3 | 2.7 | DUSTER | 1 | | 2 | | 2 | | 1.7 | |  |
| CARSON | 2 | 2 | 2 | 2.0 | E2041 | 2 | | 3 | | 2 | | 2.3 | |  |
| CENTERFIELD | 3 | 2 | 3 | 2.7 | EAGLE | 2 | | 3 | | 2 | | 2.3 | |  |
| CENTURA | 4 | 4 | 4 | 4.0 | ENHANCER | 4 | | 4 | | 4 | | 4.0 | |  |
| CENTURK78 | 3 | 3 | 3 | 3.0 | EXPEDITION | 4 | | 4 | | 4 | | 4.0 | |  |
| CENTURY | 2 | 2 | 2 | 2.0 | FULLER | 5 | | 5 | | 4 | | 4.7 | |  |
| CHENEY | 3 | 3 | 3 | 3.0 | G1878 | 3 | | 3 | | 3 | | 3.0 | |  |
| CHEYENNE | 3 | 3 | 3 | 3.0 | GAGE | 3 | | 3 | | 3 | | 3.0 | |  |
| CHISHOLM | 2 | 2 | 2 | 2.0 | GALLAGHER | 3 | | 4 | | 4 | | 3.7 | |  |
| CO03064 | 3 | 4 | 4 | 3.7 | GARRISON | 2 | | 2 | | 2 | | 2.0 | |  |
| CO03W043 | 4 | 4 | 4 | 4.0 | GENOU | 2 | | 3 | | 3 | | 2.7 | |  |
| GENT | 4 | 3 | 4 | 3.7 | LAMAR | 3 | | 2 | | 3 | | 2.7 | |  |
| GOODSTREAK | 4 | 3 | 3 | 3.3 | LANCER | 3 | | 3 | | 3 | | 3.0 | |  |
| GUYMON | 2 | 1 | 2 | 1.7 | LARNED | 3 | | 4 | | 4 | | 3.7 | |  |
| HAIL | 4 | 4 | 5 | 4.3 | LINDON | 3 | | 4 | | 4 | | 3.7 | |  |
| HALLAM | 5 | 4 | 4 | 4.3 | LONGHORN | 5 | | 5 | | 4 | | 4.7 | |  |
| HALT | 4 | 5 | 4 | 4.3 | MACE | 3 | | 4 | | 4 | | 3.7 | |  |
| HARDING | 5 | 4 | 4 | 4.3 | MCGILL | 4 | | 3 | | 3 | | 3.3 | |  |
| HARRY | 5 | 4 | 4 | 4.3 | MILLENNIUM | 4 | | 4 | | 4 | | 4.0 | |  |
| HATCHER | 4 | 4 | 5 | 4.3 | MIT | 4 | | 4 | | 4 | | 4.0 | |  |
| HEYNE | 3 | 3 | 3 | 3.0 | MT0495 | 1 | | 2 | | 2 | | 1.7 | |  |
| HG-9 | 5 | 4 | 5 | 4.7 | MT06103 | 3 | | 3 | | 3 | | 3.0 | |  |
| HOMESTEAD | 3 | 3 | 3 | 3.0 | MT85200 | 3 | | 4 | | 4 | | 3.7 | |  |
| HONDO | 4 | 4 | 5 | 4.3 | MT9513 | 3 | | 3 | | 3 | | 3.0 | |  |
| HUME | 3 | 3 | 3 | 3.0 | MT9904 | 2 | | 2 | | 2 | | 2.0 | |  |
| HV906-865 | 4 | 4 | 5 | 4.3 | MT9982 | 2 | | 2 | | 2 | | 2.0 | |  |
| HV9W03-1379R | 3 | 3 | 4 | 3.3 | MTS0531 | 2 | | 3 | | 3 | | 2.7 | |  |
| HV9W03-1551WP | 3 | 3 | 3 | 3.0 | NE02558 | 4 | | 4 | | 3 | | 3.7 | |  |
| HV9W03-1596R | 4 | 4 | 4 | 4.0 | NE04490 | 5 | | 4 | | 5 | | 4.7 | |  |
| HV9W05-1280R | 4 | 4 | 4 | 4.0 | NE05430 | 5 | | 4 | | 4 | | 4.3 | |  |
| HV9W06-504 | 1 | 2 | 2 | 1.7 | NE05496 | 2 | | 3 | | 3 | | 2.7 | |  |
| INFINITY_CL | 4 | 4 | 4 | 4.0 | NE05548 | 4 | | 3 | | 3 | | 3.3 | |  |
| INTRADA | 2 | 1 | 2 | 1.7 | NE06545 | 3 | | 3 | | 3 | | 3.0 | |  |
| JAGALENE | 5 | 5 | 4 | 4.7 | NE06607 | 2 | | 3 | | 2 | | 2.3 | |  |
| JAGGER | 3 | 3 | 3 | 3.0 | NE99495 | 2 | | 2 | | 2 | | 2.0 | |  |
| JERRY | 4 | 4 | 4 | 4.0 | NEKOTA | 4 | | 4 | | 4 | | 4.0 | |  |
| JUDEE | 5 | 4 | 4 | 4.3 | NELL | 3 | | 3 | | 3 | | 3.0 | |  |
| JUDITH | 3 | 4 | 4 | 3.7 | NEOSHO | 4 | | 3 | | 3 | | 3.3 | |  |
| JULES | 3 | 3 | 3 | 3.0 | NEWTON | 3 | | 3 | | 4 | | 3.3 | |  |
| KARL_92 | 2 | 3 | 3 | 2.7 | NI06736 | 2 | | 3 | | 3 | | 2.7 | |  |
| KAW61 | 2 | 2 | 2 | 2.0 | NI06737 | 4 | | 3 | | 3 | | 3.3 | |  |
| KEOTA | 3 | 3 | 3 | 3.0 | NI07703 | 3 | | 2 | | 2 | | 2.3 | |  |
| KHARKOF | 5 | 4 | 5 | 4.7 | NI08707 | 3 | | 3 | | 3 | | 3.0 | |  |
| KIOWA | 3 | 4 | 4 | 3.7 | NI08708 | 3 | | 4 | | 4 | | 3.7 | |  |
| KIRWIN | 3 | 4 | 4 | 3.7 | NIOBRARA | 4 | | 4 | | 4 | | 4.0 | |  |
| KS00F5-20-3 | 4 | 4 | 3 | 3.7 | NORKAN | 4 | | 3 | | 3 | | 3.3 | |  |
| LAKIN | 4 | 4 | 4 | 4.0 | NORRIS | 4 | | 4 | | 5 | | 4.3 | |  |
| NUFRONTIER | 2 | 2 | 2 | 2.0 | OK1067274 | 3 | | 3 | | 3 | | 3.0 | |  |
| NUHORIZON | 3 | 4 | 4 | 3.7 | OK1068002 | 4 | | 3 | | 3 | | 3.3 | |  |
| NUPLAINS | 4 | 4 | 4 | 4.0 | OK1068009 | 4 | | 5 | | 5 | | 4.7 | |  |
| NUSKY | 4 | 3 | 4 | 3.7 | OK1068026 | 3 | | 3 | | 3 | | 3.0 | |  |
| NW03666 | 4 | 5 | 5 | 4.7 | OK1068112 | 4 | | 4 | | 4 | | 4.0 | |  |
| OGALLALA | 4 | 4 | 5 | 4.3 | OK1070267 | 3 | | 3 | | 3 | | 3.0 | |  |
| OK_RISING | 1 | 1 | 1 | 1.0 | OK1070275 | 3 | | 3 | | 3 | | 3.0 | |  |
| OK02405 | 3 | 3 | 2 | 2.7 | ONAGA | 3 | | 4 | | 4 | | 3.7 | |  |
| OK04111 | 3 | 4 | 3 | 3.3 | OVERLAND | 4 | | 3 | | 4 | | 3.7 | |  |
| OK04415 | 4 | 3 | 4 | 3.7 | OVERLEY | 4 | | 3 | | 4 | | 3.7 | |  |
| OK04505 | 3 | 3 | 3 | 3.0 | PARKER | 3 | | 4 | | 4 | | 3.7 | |  |
| OK04507 | 4 | 3 | 3 | 3.3 | PARKER76 | 2 | | 3 | | 3 | | 2.7 | |  |
| OK04525 | 2 | 1 | 2 | 1.7 | PETE | 2 | | 2 | | 2 | | 2.0 | |  |
| OK05108 | 3 | 4 | 3 | 3.3 | PLATTE | 5 | | 5 | | 4 | | 4.7 | |  |
| OK05122 | 1 | 1 | 2 | 1.3 | POSTROCK | 3 | | 3 | | 3 | | 3.0 | |  |
| OK05134 | 4 | 4 | 4 | 4.0 | PRAIRIE_RED | 4 | | 4 | | 3 | | 3.7 | |  |
| OK05204 | 2 | 2 | 2 | 2.0 | PRONGHORN | 5 | | 4 | | 4 | | 4.3 | |  |
| OK05303 | 4 | 4 | 4 | 4.0 | PROWERS | 4 | | 4 | | 5 | | 4.3 | |  |
| OK05312 | 3 | 3 | 3 | 3.0 | RAWHIDE | 5 | | 4 | | 4 | | 4.3 | |  |
| OK05511 | 3 | 3 | 3 | 3.0 | REDLAND | 5 | | 4 | | 5 | | 4.7 | |  |
| OK05526 | 3 | 2 | 3 | 2.7 | RIPPER | 3 | | 4 | | 3 | | 3.3 | |  |
| OK05711W | 2 | 2 | 2 | 2.0 | RITA | 2 | | 3 | | 3 | | 2.7 | |  |
| OK05723W | 1 | 2 | 1 | 1.3 | ROBIDOUX | 4 | | 4 | | 5 | | 4.3 | |  |
| OK05830 | 2 | 2 | 2 | 2.0 | RONL | 3 | | 3 | | 3 | | 3.0 | |  |
| OK06114 | 4 | 3 | 4 | 3.7 | ROSE | 3 | | 3 | | 3 | | 3.0 | |  |
| OK06210 | 4 | 3 | 3 | 3.3 | ROSEBUD | 3 | | 3 | | 4 | | 3.3 | |  |
| OK06318 | 4 | 4 | 4 | 4.0 | SAGE | 3 | | 2 | | 3 | | 2.7 | |  |
| OK06319 | 3 | 4 | 3 | 3.3 | SANDY | 4 | | 4 | | 4 | | 4.0 | |  |
| OK06336 | 4 | 4 | 4 | 4.0 | SANTA_FE | 2 | | 2 | | 2 | | 2.0 | |  |
| OK07231 | 2 | 3 | 3 | 2.7 | SCOUT66 | 4 | | 5 | | 4 | | 4.3 | |  |
| OK07S117 | 3 | 3 | 3 | 3.0 | SD00111-9 | 4 | | 4 | | 4 | | 4.0 | |  |
| OK08328 | 3 | 4 | 4 | 3.7 | SD01058 | 2 | | 3 | | 3 | | 2.7 | |  |
| OK09634 | 2 | 3 | 2 | 2.3 | SD01237 | 3 | | 3 | | 3 | | 3.0 | |  |
| OK101 | 1 | 2 | 1 | 1.3 | SD05118 | 3 | | 3 | | 3 | | 3.0 | |  |
| OK10119 | 4 | 4 | 4 | 4.0 | SD05210 | 3 | | 3 | | 3 | | 3.0 | |  |
| OK102 | 2 | 3 | 2 | 2.3 | SETTLER_CL | 5 | | 4 | | 4 | | 4.3 | |  |
| OK1067071 | 4 | 3 | 4 | 3.7 | SHAWNEE | 2 | | 3 | | 3 | | 2.7 | |  |
| SHOCKER | 4 | 4 | 3 | 3.7 | TX03A0563 | 2 | | 3 | | 3 | | 2.7 | |  |
| SIOUXLAND | 4 | 4 | 4 | 4.0 | TX04A001246 | 4 | | 3 | | 3 | | 3.3 | |  |
| SMOKYHILL | 4 | 4 | 4 | 4.0 | TX04M410211 | 3 | | 4 | | 3 | | 3.3 | |  |
| SPARTAN | 5 | 4 | 4 | 4.3 | TX04V075080 | 2 | | 3 | | 3 | | 2.7 | |  |
| STANTON | 3 | 3 | 4 | 3.3 | TX05A001188 | 4 | | 3 | | 3 | | 3.3 | |  |
| STURDY | 3 | 4 | 5 | 4.0 | TX05A001822 | 4 | | 4 | | 4 | | 4.0 | |  |
| STURDY_2K | 4 | 5 | 4 | 4.3 | TX05V7259 | 3 | | 3 | | 3 | | 3.0 | |  |
| TAM105 | 3 | 3 | 3 | 3.0 | TX05V7269 | 4 | | 4 | | 4 | | 4.0 | |  |
| TAM107 | 3 | 3 | 3 | 3.0 | TX06A001132 | 4 | | 4 | | 3 | | 3.7 | |  |
| TAM107-R7 | 5 | 4 | 4 | 4.3 | TX06A001263 | 3 | | 2 | | 3 | | 2.7 | |  |
| TAM109 | 2 | 3 | 3 | 2.7 | TX06A001281 | 2 | | 2 | | 3 | | 2.3 | |  |
| TAM110 | 3 | 3 | 3 | 3.0 | TX06A001386 | 3 | | 3 | | 3 | | 3.0 | |  |
| TAM111 | 3 | 3 | 3 | 3.0 | TX06V7266 | 2 | | 2 | | 3 | | 2.3 | |  |
| TAM112 | 3 | 2 | 3 | 2.7 | TX07A001279 | 4 | | 4 | | 4 | | 4.0 | |  |
| TAM200 | 3 | 4 | 3 | 3.3 | TX07A001318 | 3 | | 3 | | 3 | | 3.0 | |  |
| TAM202 | 4 | 4 | 4 | 4.0 | TX07A001420 | 2 | | 3 | | 3 | | 2.7 | |  |
| TAM203 | 4 | 4 | 4 | 4.0 | TX86A5606 | 4 | | 5 | | 5 | | 4.7 | |  |
| TAM302 | 3 | 4 | 4 | 3.7 | TX86A6880 | 3 | | 2 | | 3 | | 2.7 | |  |
| TAM303 | 4 | 3 | 4 | 3.7 | TX86A8072 | 2 | | 3 | | 2 | | 2.3 | |  |
| TAM304 | 4 | 3 | 3 | 3.3 | TX96D1073 | 4 | | 4 | | 4 | | 4.0 | |  |
| TAM400 | 2 | 3 | 4 | 3.0 | TX99A0153-1 | 3 | | 4 | | 4 | | 3.7 | |  |
| TAM401 | 3 | 2 | 5 | 3.3 | TX99U8618 | 4 | | 4 | | 4 | | 4.0 | |  |
| TAMW-101 | 4 | 4 | 4 | 4.0 | VENANGO | 2 | | 2 | | 2 | | 2.0 | |  |
| TANDEM | 4 | 4 | 3 | 3.7 | VISTA | 4 | | 3 | | 4 | | 3.7 | |  |
| TARKIO | 5 | 4 | 5 | 4.7 | VONA | 3 | | 4 | | 4 | | 3.7 | |  |
| TASCOSA | 3 | 3 | 3 | 3.0 | W04-417 | 4 | | 4 | | 4 | | 4.0 | |  |
| THUNDER_CL | 5 | 4 | 4 | 4.3 | WAHOO | 4 | | 4 | | 4 | | 4.0 | |  |
| THUNDERBOLT | 4 | 3 | 4 | 3.7 | WARRIOR | 5 | | 5 | | 5 | | 5.0 | |  |
| TREGO | 3 | 2 | 3 | 2.7 | WB411W | 3 | | 2 | | 3 | | 2.7 | |  |
| TRISON | 2 | 2 | 2 | 2.0 | WENDY | 5 | | 4 | | 4 | | 4.3 | |  |
| TRIUMPH64 | 2 | 3 | 2 | 2.3 | WESLEY | 3 | | 3 | | 3 | | 3.0 | |  |
| TURKEY_NEBSEL | 3 | 3 | 3 | 3.0 | WICHITA | 5 | | 4 | | 4 | | 4.3 | |  |
| TX00V1131 | 4 | 4 | 3 | 3.7 | WINDSTAR | 4 | | 4 | | 4 | | 4.0 | |  |
| TX01A5936 | 3 | 4 | 4 | 3.7 | WINOKA | 2 | | 3 | | 3 | | 2.7 | |  |
| TX01M5009-28 | 1 | 2 | 2 | 1.7 | YELLOWSTONE | 2 | | 3 | | 3 | | 2.7 | |  |
| TX01V5134RC-3 | 2 | 3 | 3 | 2.7 | YUMA | 2 | | 2 | | 2 | | 2.0 | |  |
| TX02A0252 | 2 | 4 | 3 | 3.0 | YUMAR | 1 | | 2 | | 2 | | 1.7 | |  |
| TX03A0148 | 3 | 4 | 4 | 3.7 |  | |  | |  | |  | |  | |
