# Supplementary material for: Genome-Wide Association Study for Spot Blotch Resistance in Hard Winter Wheat
Source: Front Plant Sci. 2018 Jul 6;9:926. doi: 10.3389/fpls.2018.00926 (PMC6043670; doi:10.3389/fpls.2018.00926)
Supplement: Supplementary file 2 [file Table_2.docx]

Supplementary Table 2. Linear mixed model analysis of spot blotch scores recorded from 294 genotypes of the hard winter wheat association mapping panel (HWWAMP).

| **Effects** | **Estimate** | **SE** | **P-value** | **2.5%LL** | **97.5%UL** |
| --- | --- | --- | --- | --- | --- |
| Genotype | 0.8040 | 0.0077 | 1.33E-14 | 0.7768 | 0.8311 |
| Experiment | 0.0011 | 0.0009 | 6.24E-01 | -0.0020 | 0.0042 |
| Genotypes*Experiment | 0.0975 | 0.0042 | 8.88E-09 | 0.0828 | 0.1121 |
| Residual error | 0.0975 | 0.0042 | 8.88E-09 | 0.0828 | 0.1121 |
| **Proportion of effects** |  | | | | |
| V(Genotype)/VP | 0.5797 | 0.0114 | 9.17E-12 | 0.5392 | 0.6201 |
| V(Experiment)/VP | 0.0008 | 0.0006 | 6.25E-01 | -0.0014 | 0.0031 |
| V(Genotype*Experiment)/VP | 0.0702 | 0.0026 | 2.51E-09 | 0.0611 | 0.0794 |
| V(e)/VP | 0.0702 | 0.0026 | 2.51E-09 | 0.0611 | 0.0794 |
| **Heritability & repeatability** |  | | | | |
| Broad-sense heritability, H^2^ (%) | 80.4 | | | | |
| Repeatability (%) | 96.1 | | | | |

VP - total variance; SE - slandered error; LL - lower limit; UL - upper limit
