# Supplementary material for: Genome-Wide Association Study for Spot Blotch Resistance in Hard Winter Wheat
Source: Front Plant Sci. 2018 Jul 6;9:926. doi: 10.3389/fpls.2018.00926 (PMC6043670; doi:10.3389/fpls.2018.00926)
Supplement: Supplementary file 3 [file Table_3.docx]

Supplementary Table 3. Summary of number, minor allele frequency (MAF) and density of markers used for spot blotch GWAS in 294 wheat genotypes of hard winter wheat association mapping panel (HWWAMP).

| **Chromosomes** | | **No. of markers** | **Chromosome length (cM)** | **Minor allele frequency** | **Average number of marker/cM** | **Average distance between**  **markers (cM)** | |
| --- | --- | --- | --- | --- | --- | --- | --- |
| Genome A  1A-7A | 1 | 1036 | 161.335 | 0.2419 | 6.4 | 0.16 |  |
|  | 2 | 956 | 179.116 | 0.1998 | 5.3 | 0.19 |  |
|  | 3 | 773 | 188.378 | 0.2442 | 4.1 | 0.24 |  |
|  | 4 | 744 | 164.13 | 0.1889 | 4.5 | 0.22 |  |
|  | 5 | 790 | 148.304 | 0.2333 | 5.3 | 0.19 |  |
|  | 6 | 958 | 164.089 | 0.2093 | 5.8 | 0.17 |  |
|  | 7 | 954 | 244.155 | 0.2417 | 3.9 | 0.26 |  |
|  | - | 887**†** | 178.501**†** | 0.2227† | 5.0**†** | 0.20**†** |  |
| Genome B  1B-7B | 1 | 1254 | 173.624 | 0.2415 | 7.2 | 0.14 |  |
|  | 2 | 1162 | 185.666 | 0.2485 | 6.3 | 0.16 |  |
|  | 3 | 1117 | 149.634 | 0.2727 | 7.5 | 0.13 |  |
|  | 4 | 451 | 119.446 | 0.2591 | 3.8 | 0.26 |  |
|  | 5 | 1554 | 219.773 | 0.2100 | 7.1 | 0.14 |  |
|  | 6 | 1237 | 127.049 | 0.2620 | 9.7 | 0.10 |  |
|  | 7 | 855 | 178.856 | 0.2259 | 4.8 | 0.21 |  |
|  | - | 1090 **†** | 164.864**†** | 0.2457† | 6.6**†** | 0.16**†** |  |
| Genome D  1D-7D | 1 | 438 | 192.274 | 0.1939 | 2.3 | 0.44 |  |
|  | 2 | 581 | 138.737 | 0.1688 | 4.2 | 0.24 |  |
|  | 3 | 240 | 164.672 | 0.2176 | 1.5 | 0.69 |  |
|  | 4 | 52 | 170.428 | 0.2205 | 0.3 | 3.28 |  |
|  | 5 | 159 | 207.329 | 0.2580 | 0.8 | 1.30 |  |
|  | 6 | 146 | 160.498 | 0.2517 | 0.9 | 1.10 |  |
|  | 7 | 133 | 223.524 | 0.2662 | 0.6 | 1.68 |  |
|  | - | 250**†** | 179.637**†** | 0.2252† | 1.5**†** | 1.25**†** |  |
| Total | - | 742 | 174.334 | 0.2312 | 4.4 | 0.54 |  |

† corresponding mean values of genomes A, B, and D
