# Supplementary material for: Genome-Wide Association Study for Spot Blotch Resistance in Hard Winter Wheat
Source: Front Plant Sci. 2018 Jul 6;9:926. doi: 10.3389/fpls.2018.00926 (PMC6043670; doi:10.3389/fpls.2018.00926)
Supplement: Supplementary file 4 [file Table_4.docx]

Supplementary Table 4. Syntenic and non-syntenic LD for the whole, A, B, and D genomes of winter wheat lines in hard winter wheat association mapping panel (HWWAMP).

| Dataset | Number of markers | Total marker pairs | Mean of r^2^ for all pairs | Total unlinked pairs | Significant pairs (P<0.001) | | |
| --- | --- | --- | --- | --- | --- | --- | --- |
|  |  |  |  |  | **Total**† | **Linked**  **(r^2^ >0.1)** | **Unlinked**  **(r^2^ <0.1)** |
| Whole genome | 1,842 | 91,307 | 0.03 | 85,955 | 13,076 (14.3) | 5,332 (5.8) | 7,744 (8.5) |
| A | 739 | 39,216 | 0.027 | 37,065 | 5,225 (13.3) | 2,151  ( 5.5) | 3,074 (7.8) |
| B | 782 | 43,995 | 0.028 | 41,491 | 6,489 (14.7) | 2,504  (5.7) | 3,985 (9.1) |
| D | 321 | 8,096 | 0.05 | 7,399 | 1,382 (17.1) | 6,97  (8.6) | 685  (8.5) |

† Percent of markers out of total markers in parenthesis
