# Supplementary material for: Genome-Wide Association Study for Spot Blotch Resistance in Hard Winter Wheat
Source: Front Plant Sci. 2018 Jul 6;9:926. doi: 10.3389/fpls.2018.00926 (PMC6043670; doi:10.3389/fpls.2018.00926)
Supplement: Supplementary file 5 [file Table_5.docx]

Supplementary Table 5. Comparative analysis of the significant SNPs associated with spot blotch resistance detected by MLM and GLM in hard winter wheat association mapping panel (HWWAMP).

| **Number of Significant Markers** | **MLM** | | **GLM** | |
| --- | --- | --- | --- | --- |
|  | **P<0.01** | **P<0.001** | **P<0.01** | **P<0.001** |
| Total significant markers | 117 | 13 | 198 | 26 |
| Shared marker | 77 | 13 | 77 | 13 |
| Unique significant marker | 40 | 0 | 121 | 13 |
| % shared marker | 66 | 100 | 39 | 50 |
| % Unique significant marker | 34 | 0 | 51 | 50 |
