# Supplementary material for: Genome-Wide Association Study for Spot Blotch Resistance in Hard Winter Wheat
Source: Front Plant Sci. 2018 Jul 6;9:926. doi: 10.3389/fpls.2018.00926 (PMC6043670; doi:10.3389/fpls.2018.00926)
Supplement: Supplementary file 6 [file Table_6.docx]

Supplementary Table 6. SNPs associated with spot blotch resistance identified through GWAS in the 294 genotypes of hard winter wheat association mapping panel (HWWAMP).

| **Markers** | **Chr.** | **Pos. (cM)** | **P-value** | **R2** | **Allele 1** | **Allele 2** | **Additive effects*** | **Genotypes with allele type** | |
| --- | --- | --- | --- | --- | --- | --- | --- | --- | --- |
|  |  |  |  |  |  |  |  | **1** | **2** |
| Tdurum_contig29769_202 | 1B | 64.5 | 4.18E-03 | 0.03 | A | G | -0.47 | 35 | 258 |
| Kukri_rep_c111517_289 | 1B | 64.9 | 4.15E-03 | 0.03 | C | T | 0.47 | 257 | 35 |
| Excalibur_c51643_145 | 1B | 64.9 | 4.18E-03 | 0.03 | C | T | -0.47 | 35 | 258 |
| Excalibur_rep_c106174_390 | 1B | 64.9 | 4.47E-03 | 0.03 | C | T | 0.46 | 252 | 35 |
| CAP7_c1241_128 | 1B | 64.9 | 4.88E-03 | 0.03 | A | G | -0.46 | 35 | 259 |
| Excalibur_c27675_912 | 1B | 64.9 | 4.88E-03 | 0.03 | A | G | 0.46 | 259 | 35 |
| Excalibur_c32608_500 | 1B | 64.9 | 4.88E-03 | 0.03 | A | G | -0.46 | 35 | 259 |
| Kukri_rep_c106406_265 | 1B | 64.9 | 4.88E-03 | 0.03 | C | T | -0.46 | 35 | 259 |
| BS00022104_51 | 1B | 64.9 | 5.04E-03 | 0.03 | A | G | -0.46 | 35 | 257 |
| IWA7040 | 1B | 84.4 | 3.05E-03 | 0.03 | A | C | -0.41 | 214 | 78 |
| IWA4940 | 1B | 84.4 | 3.11E-03 | 0.03 | A | G | 0.41 | 77 | 214 |
| IACX4411 | 1B | 108 | 4.08E-03 | 0.03 | A | G | 0.35 | 190 | 99 |
| IWA5749 | 1B | 108.4 | 3.91E-03 | 0.03 | C | T | -0.34 | 102 | 192 |
| BobWhite_c20621_541 | 1B | 108.4 | 3.91E-03 | 0.03 | C | T | -0.34 | 102 | 192 |
| Excalibur_c15692_532 | 1D | 33 | 4.79E-03 | 0.03 | G | T | 0.31 | 140 | 150 |
| IWA5574 | 2A | 113.3 | 4.04E-03 | 0.03 | C | T | 0.38 | 72 | 218 |
| IACX6337 | 2A | 113.3 | 4.07E-03 | 0.03 | A | G | -0.38 | 222 | 72 |
| IWA5449 | 2A | 113.3 | 4.09E-03 | 0.03 | C | T | -0.38 | 219 | 72 |
| Kukri_c27100_823 | 2A | 113.3 | 4.09E-03 | 0.03 | C | T | 0.38 | 72 | 219 |
| Excalibur_c64568_149 | 2A | 145.3 | 1.81E-03 | 0.03 | C | T | -0.66 | 18 | 272 |
| Kukri_c25281_99 | 2B | 102.5 | 4.49E-03 | 0.03 | A | G | 0.33 | 199 | 90 |
| Kukri_c31121_1460 | 2D | 80.1 | 9.87E-04 | 0.04 | C | T | -0.45 | 54 | 238 |
| RAC875_c4851_1600 | 2D | 103.3 | 2.37E-03 | 0.03 | C | T | -0.62 | 21 | 273 |
| IAAV9128 | 2D | 103.3 | 2.59E-03 | 0.03 | A | G | 0.61 | 271 | 21 |
| Excalibur_c46082_440 | 3A | 90.5 | 9.97E-04 | 0.04 | C | T | -0.37 | 166 | 123 |
| IAAV2383 | 3A | 90.6 | 8.39E-04 | 0.04 | A | G | 0.37 | 126 | 163 |
| Kukri_c11709_874 | 3A | 109.9 | 3.71E-03 | 0.03 | C | T | -0.52 | 255 | 31 |
| BS00049637_51 | 3A | 109.9 | 3.77E-03 | 0.03 | C | T | 0.52 | 31 | 260 |
| BS00009440_51 | 3B | 51.1 | 3.30E-03 | 0.03 | A | G | -0.33 | 94 | 200 |
| GENE-1167_104 | 3B | 80.1 | 3.70E-03 | 0.03 | A | G | 0.48 | 36 | 255 |
| Kukri_c43208_335 | 3D | 67.2 | 3.61E-03 | 0.03 | A | G | -0.4 | 230 | 61 |
| IWA4209 | 3D | 67.2 | 4.03E-03 | 0.03 | A | G | 0.39 | 64 | 230 |
| IWA8179 | 3D | 107.9 | 4.06E-03 | 0.03 | A | G | -0.4 | 229 | 62 |
| BobWhite_c17386_221 | 3D | 107.9 | 4.43E-03 | 0.03 | C | T | -0.39 | 230 | 62 |
| Excalibur_c24600_733 | 4A | 114.5 | 2.45E-03 | 0.03 | C | T | 0.36 | 80 | 210 |
| Ra_c16330_1197 | 4A | 114.5 | 2.53E-03 | 0.03 | A | G | -0.36 | 212 | 82 |
| Excalibur_c14217_1260 | 4A | 114.5 | 2.81E-03 | 0.03 | C | T | -0.36 | 209 | 80 |
| TA004912-0408 | 4A | 114.5 | 3.20E-03 | 0.03 | C | T | -0.35 | 213 | 80 |
| IWA8475 | 4A | 118.7 | 1.55E-04 | 0.05 | G | T | -0.44 | 204 | 90 |
| BS00064369_51 | 4A | 118.7 | 9.10E-04 | 0.04 | A | C | -0.39 | 209 | 84 |
| BS00039641_51 | 4A | 125.9 | 2.56E-03 | 0.03 | A | G | 0.46 | 244 | 47 |
| BS00022998_51 | 4A | 127.1 | 1.89E-03 | 0.03 | C | T | -0.47 | 46 | 242 |
| BS00000577_51 | 4A | 127.1 | 2.83E-03 | 0.03 | C | T | 0.44 | 241 | 49 |
| GENE-0689_791 | 4A | 127.1 | 3.20E-03 | 0.03 | A | G | 0.44 | 239 | 49 |
| GENE-0689_776 | 4A | 127.1 | 4.09E-03 | 0.03 | C | T | -0.42 | 50 | 242 |
| IWA3864 | 4A | 127.1 | 4.09E-03 | 0.03 | A | G | -0.42 | 50 | 242 |
| BS00084703_51 | 4A | 127.1 | 4.37E-03 | 0.03 | A | G | 0.43 | 242 | 48 |
| BS00111091_51 | 4A | 127.1 | 4.62E-03 | 0.03 | A | G | -0.42 | 50 | 241 |
| BS00099982_51 | 4A | 127.1 | 4.91E-03 | 0.03 | G | T | 0.41 | 244 | 50 |
| Excalibur_c15222_313 | 4A | 132.9 | 4.98E-03 | 0.03 | C | T | 0.45 | 246 | 39 |
| Excalibur_c11302_186 | 4A | 135.2 | 1.43E-03 | 0.04 | C | T | -0.72 | 17 | 268 |
| Excalibur_rep_c79414_306 | 4B | 36.8 | 7.30E-04 | 0.04 | A | G | 0.38 | 121 | 169 |
| GENE-4933_489 | 4B | 36.8 | 1.06E-03 | 0.04 | A | G | -0.37 | 172 | 122 |
| Tdurum_contig50625_2342 | 4B | 36.8 | 1.06E-03 | 0.04 | A | C | 0.37 | 122 | 172 |
| IACX7746 | 4B | 36.8 | 1.47E-03 | 0.04 | A | G | -0.36 | 166 | 118 |
| GENE-4933_1085 | 4B | 36.8 | 1.56E-03 | 0.04 | A | G | 0.36 | 121 | 166 |
| GENE-4933_1095 | 4B | 36.8 | 1.59E-03 | 0.04 | A | G | -0.36 | 167 | 119 |
| BS00009480_51 | 4B | 36.8 | 1.63E-03 | 0.04 | A | G | -0.36 | 169 | 118 |
| BS00009426_51 | 4B | 36.8 | 1.73E-03 | 0.03 | C | T | -0.35 | 171 | 123 |
| IACX8647 | 4B | 36.8 | 2.17E-03 | 0.03 | A | G | 0.35 | 119 | 169 |
| BS00010115_51 | 4B | 36.8 | 2.68E-03 | 0.03 | C | T | -0.34 | 169 | 123 |
| GENE-3572_70 | 5A | 46.7 | 1.33E-03 | 0.04 | C | T | 0.45 | 55 | 233 |
| Kukri_rep_c104877_2166 | 5A | 59.1 | 8.27E-05 | 0.05 | G | T | 0.66 | 257 | 35 |
| IWA6895 | 5B | 83 | 2.13E-03 | 0.03 | C | T | -0.48 | 40 | 248 |
| Excalibur_c25898_434 | 6A | 99.4 | 3.46E-03 | 0.03 | C | T | 0.6 | 274 | 20 |
| BobWhite_c20735_255 | 7B | 3 | 1.30E-03 | 0.04 | A | G | 0.4 | 77 | 209 |
| Kukri_c21628_1215 | 7B | 85.3 | 5.35E-05 | 0.06 | A | G | -0.45 | 127 | 163 |
| Excalibur_c5700_670 | 7B | 85.3 | 6.14E-05 | 0.06 | G | T | -0.44 | 127 | 167 |
| Tdurum_contig9966_646 | 7B | 86.1 | 8.09E-05 | 0.05 | A | G | -0.43 | 128 | 166 |
| TA005844-0160 | 7B | 86.4 | 4.20E-05 | 0.06 | C | T | -0.46 | 146 | 140 |
| Excalibur_c5700_705 | 7B | 86.4 | 5.05E-05 | 0.06 | A | G | 0.45 | 142 | 147 |
| Excalibur_c58742_144 | 7B | 86.4 | 5.11E-05 | 0.06 | A | C | -0.45 | 147 | 142 |
| Excalibur_c5700_527 | 7B | 86.4 | 5.35E-05 | 0.06 | C | T | -0.45 | 127 | 163 |
| Kukri_c22495_552 | 7B | 86.4 | 6.26E-05 | 0.06 | A | C | -0.44 | 131 | 163 |
| Tdurum_contig90495_232 | 7B | 87.4 | 1.35E-03 | 0.04 | A | G | 0.35 | 161 | 129 |
| BS00075332_51 | 7B | 87.4 | 1.97E-03 | 0.03 | G | T | 0.34 | 163 | 126 |

*Additive effect refers to Allele 1
