# Supplementary material for: Genome-Wide Association Study for Spot Blotch Resistance in Hard Winter Wheat
Source: Front Plant Sci. 2018 Jul 6;9:926. doi: 10.3389/fpls.2018.00926 (PMC6043670; doi:10.3389/fpls.2018.00926)
Supplement: Supplementary file 7 [file Table_7.docx]

Supplementary Table 7. Chromosome location and nucleotide sequences flanking the significant SNPs associated with spot blotch resistance.

| **Chr.** | **Markers** | **Location*** | **Marker sequence**** |
| --- | --- | --- | --- |
| 2D | Kukri_c31121_1460 | 607423370-607423456 | GGTAAAGGGGTGTCCTTCTACAGATTGTTCTGCGGCAATAGCATGTCAGA**[C/T]**GCTGCTCGACTCTTTCCCAGTGATTCAAGTGTGACCGTAGCAGGAAGCGG |
| 3A | Excalibur_c46082_440 | 556462254-556462146 | CTAGTTTTCAGCATTGGATACTTGGGTAATGGACGCTGTCTAGTTTTCTG**[C/T]**ATGTACATGTCTGTGTTCAAATAAATGCAAGGTTGGCATGTCGTACATGC |
| 4A | IWA8475 | 692383211-692383111 | TTCATCTTTGGACTGAGTTTCCCATGAAGAGGTGGATTATTGGATTGCCT**[G/T]**GTGACTCGGCTGTACTATTTTGTTAAATCGTTTGTTTCACCTACGGTTTC |
| 4B | Excalibur_rep_c79414_306 | 14118264-14118164 | TTCGAAAGAGCGTTGAAGCAGAGCCTCGAGAGGGTGCGGATCAGCGCTAG**[A/G]**TGGATCGACAGCATCAAGAGCGAGCCCAGCCTTGCGCAAACGGTGCAGCA |
| 5A | Kukri_rep_c104877_2166 | 480285174-480285274 | GAAACATGGCAGTTTCTGATGTGAAGGCTGTCATGTTGGAAATGAACACA**[G/T]**CAGATAGCGTTCAAACACAAGATCTCAAGTCGGCATCTGAAGACAGGAGT |
| 7B | TA005844-0160 | 608913624-608913673 | CTTCCCACGCATGAAACTGTACAATTTGTTACACGGATGCCAATATCCAT**[C/T]**CCT |

*The location is based on the RefSeq v1.0

**Nucleotide in the parenthesis are SNPs
