# Supplementary material for: Genome-Wide Association Study for Spot Blotch Resistance in Hard Winter Wheat
Source: Front Plant Sci. 2018 Jul 6;9:926. doi: 10.3389/fpls.2018.00926 (PMC6043670; doi:10.3389/fpls.2018.00926)
Supplement: Supplementary file 8 [file Table_8.docx]

Supplementary Table 8. Hard winter wheat genotypes that harbor multiple favorable alleles for spot blotch resistance QTLs.

| **Wheat genotype** | **Disease**  **score** | **Disease**  **reaction** | **Favorable**  **allele for QTL** | **Additive effect** | **Unfavorable**  **allele for QTL** | **Additive effect** |
| --- | --- | --- | --- | --- | --- | --- |
| Colt | 1 | R | 2D, 3A, 4A, 4B, 7B | -1.73 | 5A | 0.67 |
| Custer | 1 | R | 3A, 4A, 4B, 5A | -0.81 | 2D, 7B | 0.00 |
| OK05723W | 1 | R | 4A, 5A, 7B | -0.90 | 2D, 3A, 4B | 0.38 |
| Venango | 1 | R | 3A, 4B, 5A, 7B | -0.83 | 2D, 4A | 0.00 |
| Duster | 1 | R | 3A, 4A, 4B, 7B | -1.27 | 2D, 5A | 0.67 |

Note: - disease reducing (favorable); + disease increasing (unfavorable). Additive effects represents the sum of the corresponding favorable or unfavorable alleles.
