# Supplementary material for: Genome-Wide Association Study for Spot Blotch Resistance in Hard Winter Wheat
Source: Front Plant Sci. 2018 Jul 6;9:926. doi: 10.3389/fpls.2018.00926 (PMC6043670; doi:10.3389/fpls.2018.00926)
Supplement: Supplementary file 9 [file Table_9.docx]

Supplementary Table 9. Additive effects of alleles on resistant genotypes based on significant markers associated with spot blotch resistance.

| **SNP Markers** | **Additive effect** | **Alleles** | | **Chr.** | **Wheat Genotype** | | | | | |
| --- | --- | --- | --- | --- | --- | --- | --- | --- | --- | --- |
|  |  | **A** | **B** |  | **Colt** | **Custer** | **OK05723W** | **Venango** | **Duster** |  |
| Kukri_c31121_1460 | -0.45 | C | T | 2D | CC | TT | TT | TT | TT |  |
| Excalibur_c46082_440 | -0.37 | C | T | 3A | CC | CC | TT | CC | CC |  |
| IWA8475 | -0.44 | G | T | 4A | GG | GG | GG | TT | GG |  |
| Excalibur_rep_c79414_306 | +0.38 | A | G | 4B | GG | GG | AA | GG | GG |  |
| Kukri_rep_c104877_2166 | +0.67 | G | T | 5A | GG | TT | TT | TT | GG |  |
| TA005844-0160 | -0.46 | C | T | 7B | CC | TT | CC | CC | CC |  |

Allele A indicates additive effect; allele B indicates no effect (zero effect); The effect of allele B for a marker is always zero and the effect of allele A estimate are relative to that; - disease reducing allele; + disease increasing allele.
