# Supplementary figures and images for: Genome-Wide Association Study for Spot Blotch Resistance in Hard Winter Wheat
Source: Front Plant Sci. 2018 Jul 6;9:926. doi: 10.3389/fpls.2018.00926 (PMC6043670; doi:10.3389/fpls.2018.00926)

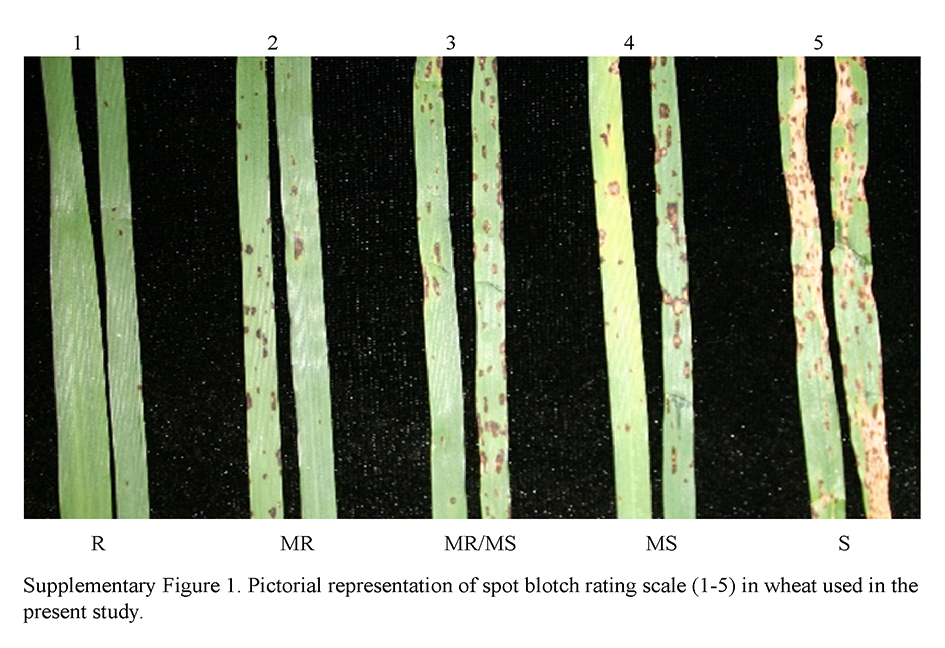

Supplement: Supplementary file 11 [file Image_1.tif]

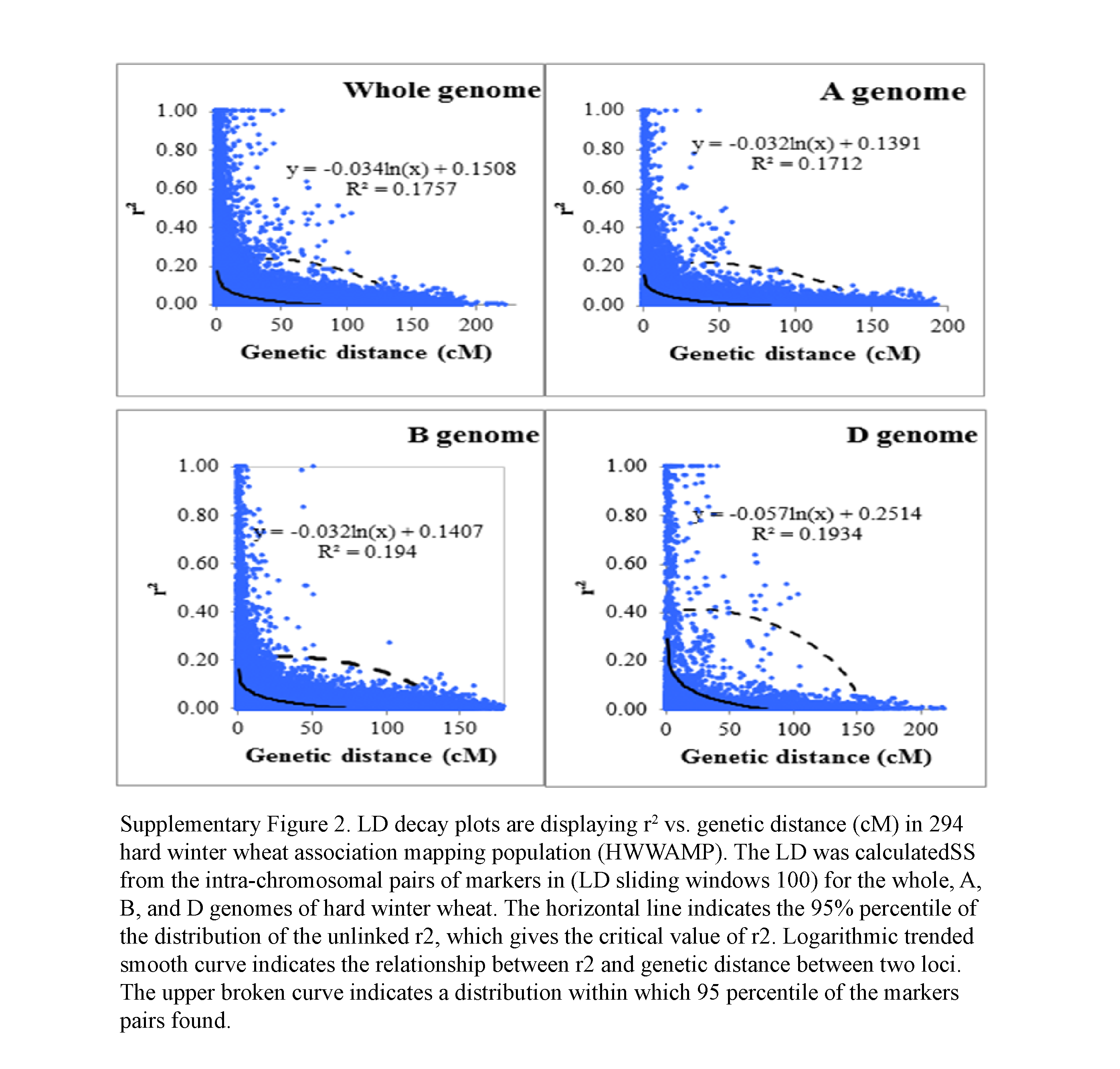

Supplement: Supplementary file 12 [file Image_2.tif]

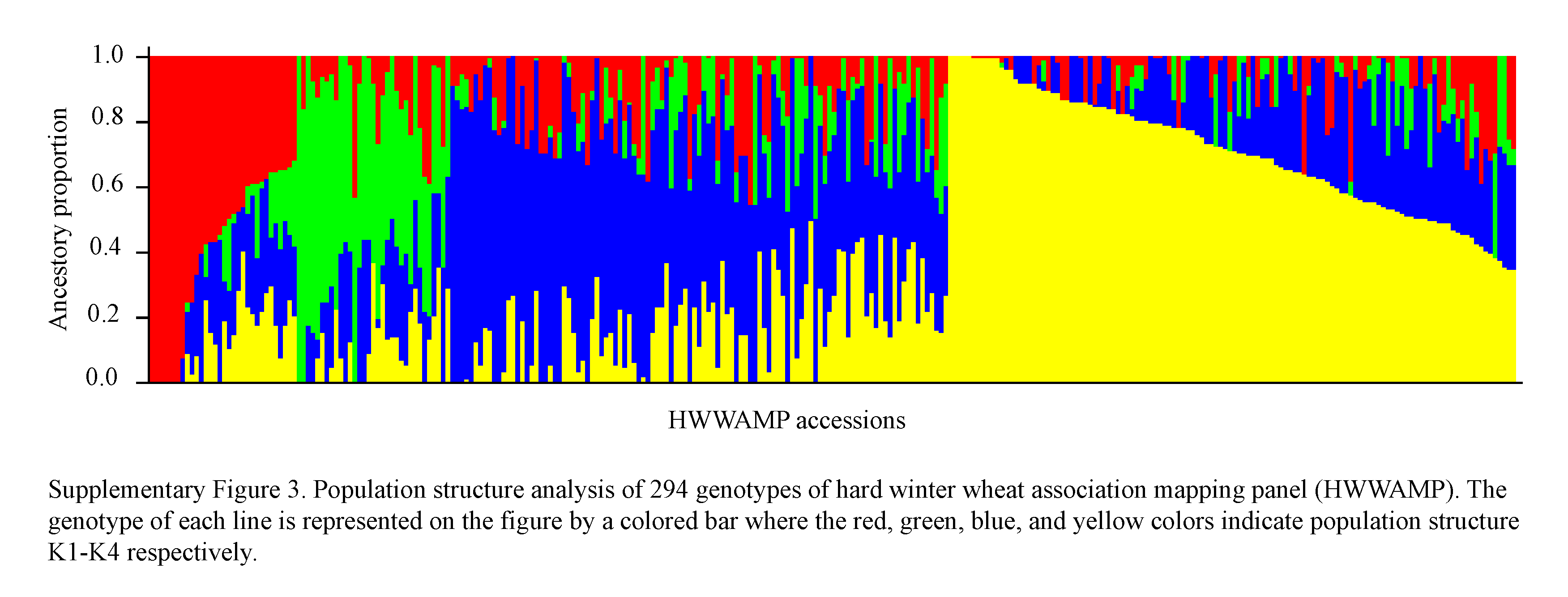

Supplement: Supplementary file 13 [file Image_3.tif]

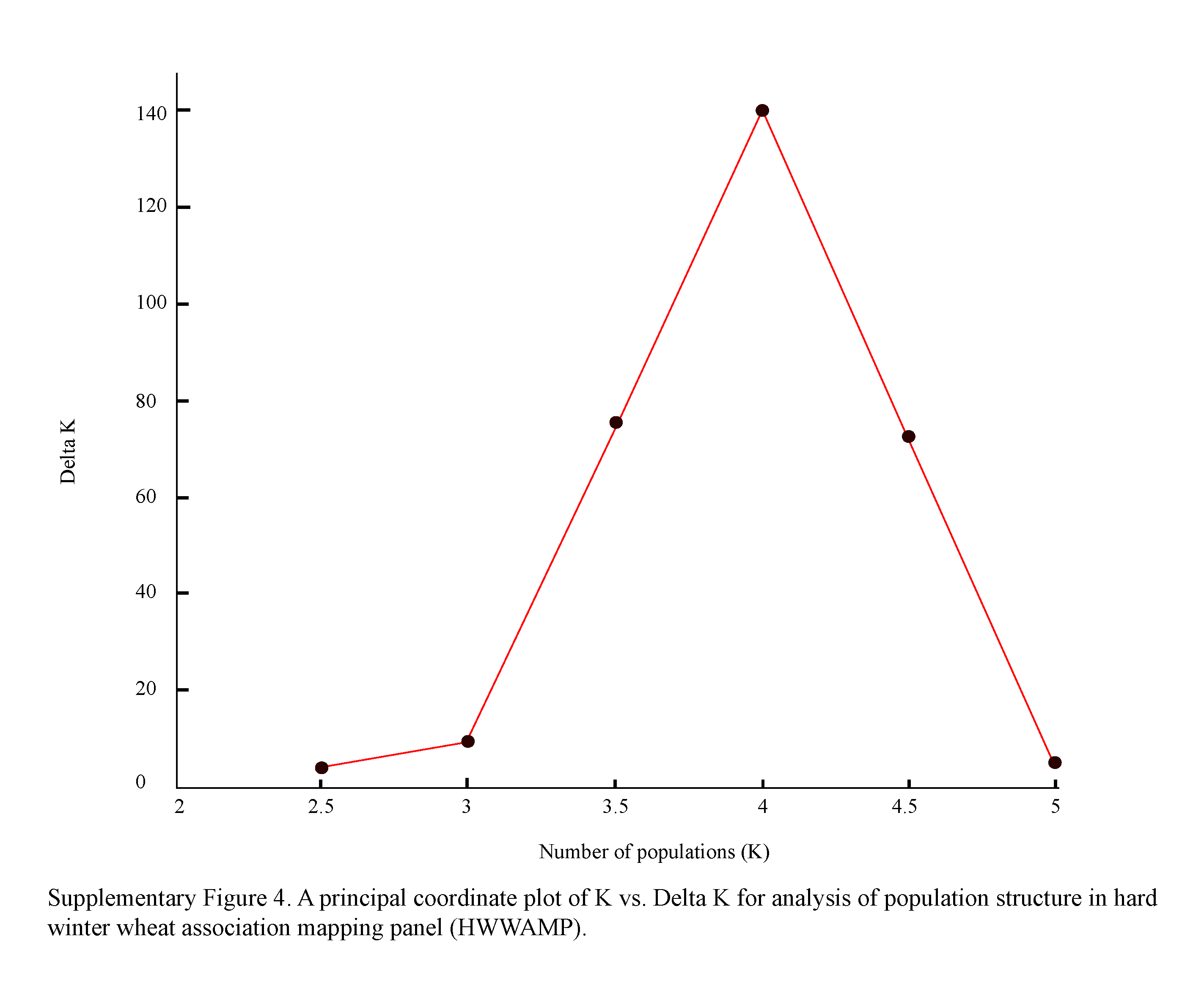

Supplement: Supplementary file 14 [file Image_4.tif]

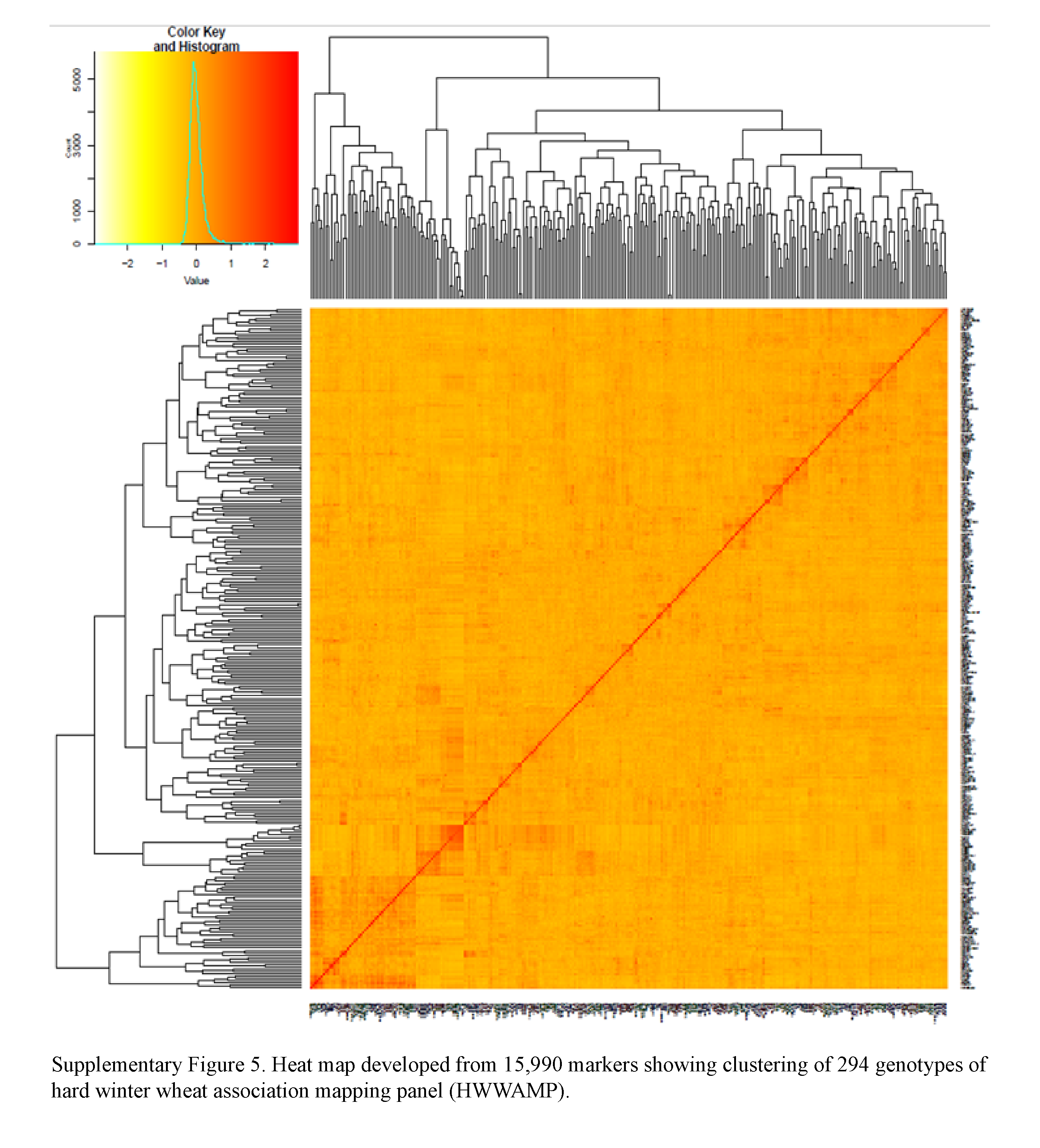

Supplement: Supplementary file 15 [file Image_5.tif]

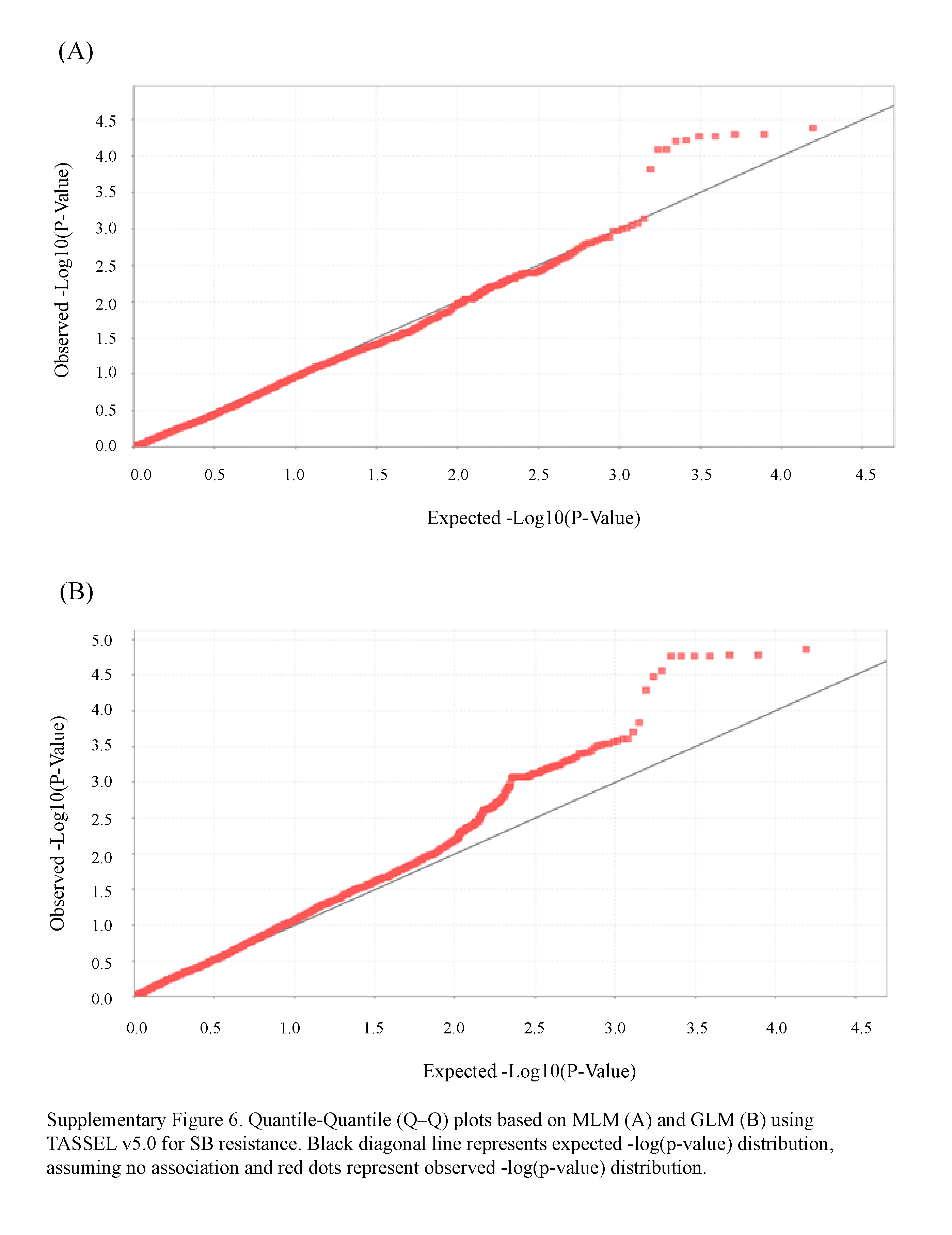

Supplement: Supplementary file 16 [file Image_6.tif]
